# Metastasis and cancer associated fibroblasts: taking it up a NOTCH

**DOI:** 10.3389/fcell.2023.1277076

**Published:** 2024-01-10

**Authors:** Argha Ghosh, Anirban K. Mitra

**Affiliations:** ^1^ Indiana University School of Medicine-Bloomington, Bloomington, IN, United States; ^2^ Indiana University Melvin and Bren Simon Comprehensive Cancer Center, Indianapolis, IN, United States; ^3^ Department of Medical and Molecular Genetics, Indiana University School of Medicine, Indianapolis, IN, United States

**Keywords:** cancer metastasis, tumor microenvironment, Notch signaling, cancer associated fibroblasts, ovarian cancer

## Abstract

Metastasis is the least understood aspect of cancer biology. 90% of cancer related deaths occur due extensive metastatic burden in patients. Apart from metastasizing cancer cells, the pro-tumorigenic and pro-metastatic role of the tumor stroma plays a crucial part in this complex process often leading to disease relapse and therapy resistance. Cellular signaling processes play a crucial role in the process of tumorigenesis and metastasis when aberrantly turned on, not just in the cancer cells, but also in the cells of the tumor microenvironment (TME). One of the most conserved pathways includes the Notch signaling pathway that plays a crucial role in the development and progression of many cancers. In addition to its well documented role in cancer cells, recent evidence suggests crucial involvement of Notch signaling in the stroma as well. This review aims to highlight the current findings focusing on the oncogenic role of notch signaling in cancer cells and the TME, with a specific focus on cancer associated fibroblasts (CAFs), which constitute a major part of the tumor stroma and are important for tumor progression. Recent efforts have focused on the development of anti-cancer and anti-metastatic therapies targeting TME. Understanding the importance of Notch signaling in the TME would help identify important drivers for stromal reprogramming, metastasis and importantly, drive future research in the effort to develop TME-targeted therapies utilizing Notch.

## Introduction

Metastasis remains the dark matter of cancer biology. Even though it is the leading cause of cancer related deaths, we have the least understanding of this intricate process and its regulation (Lengyel, 2010; Castaneda et al., 2022; Gerstberger et al., 2023). This is even more evident in cancers like that of the ovary, where the high metastatic burden in patients makes it the deadliest gynecological malignancy. Importantly, even though several key molecular drivers for its regulation have been identified, no current anti-metastatic therapy has been approved for the clinic. This further reduces survival expectancy of patients who have been diagnosed with metastasized tumors. Therefore, there is an urgent need to understand and unravel the complex mechanisms driving metastasis, which will help design effective therapies.

Over the past decades, it has become increasingly clear that metastasis is not just driven by the metastasizing cancer cells but actively involves multiple components of the tumor microenvironment (TME). The TME comprises mostly of stomal cells including endothelial cells (ECs), cancer associated fibroblasts (CAFs), adipocytes, populations of immune cells, mesothelial cells, among others that have been reprogrammed by the cancer cells to aid in tumor progression and metastasis (Quail and Joyce, 2013; Bussard et al., 2016; Baghban et al., 2020; de Visser and Joyce, 2023). A major part of the establishment of successful metastases involves an adaptation process undertaken by the cancer cells. This adaptation is critical for the successful survival and establishment of metastatic tumors in this foreign microenvironment of the metastatic site. The critical step in this process involves the crosstalk and productive interactions with and subsequent reprogramming of the indolent normal microenvironment to a more supportive and activated TME capable of hosting and benefiting the metastatic cancer cells (Mitra et al., 2012).

Studies from various cancers have indicated the metastatic TME to be highly desmoplastic, defined by significant enrichment of CAFs and extracellular matrix (ECM) components (∼10%–60%) (Piersma et al., 2020; Zeltz et al., 2020). This indicates a major role of these stromal cells in mediating metastatic colonization and progression in cancer. CAFs have also been recognized as important players in the tumorigenic progression of various cancers such as breast, lung, colorectal, ovarian, etc. (Sahai et al., 2020; Biffi and Tuveson, 2021; Chen et al., 2021). CAFs mediate tumorigenesis and metastasis largely by secretion of various paracrine factors and growth factors such as VEGF, PDGF, HGF (Chen et al., 2021; Chhabra and Weeraratna, 2023). They can also module immune functions by secretion of various cytokines and interleukins such as, IL-6, IL-8, IL-4 etc. Moreover, recent evidence points to the importance of CAFs in modulating the metabolic landscape in cancer cells, by production and secretion of metabolites such as lactate, fatty acids, and various amino acids (Becker et al., 2020; Sahai et al., 2020; Li et al., 2021; Sazeides and Le, 2021). However, the most important role of CAFs in respect to the TME is in the remodeling of the extracellular matrix (ECM). CAFs can do so both by the secretion of various ECM proteins such as fibronectin, collagens I, IV, and by the secretion of various proteases, including matrix metalloproteases (MMPs) such as MMP1, 3 and 9, urokinase, etc., that degrade the ECM (Piersma et al., 2020; Sahai et al., 2020; Chen et al., 2021). This constant degradation and rebuilding of the ECM by the CAFs are critical for the establishment of new metastases, invasion, and proliferation of the metastasizing cancer cells. However, a big question of how normal fibroblasts (NFs) or mesenchymal stem cells are recruited and converted into CAFs still remains to be elicited in detail.

Recent studies on the TME have largely focused on identifying key molecular mechanisms and factors that mediate the conversion of NFs to CAFs (Albrengues et al., 2015; Becker et al., 2020; Xue et al., 2021). It is very interesting to note that most of the driving forces and signals that play role in the recruitment of CAFs and conversion of NFs to CAFs come from the cancer cells (Malanchi et al., 2011). Cancer cells do so by secreting cellular factors and miRNAs such as, TGFB, lysophosphatidic acid, osteopontin, miR-105, miR-155, etc., that are important in this process (Pang et al., 2015; Sharon et al., 2015; Yan et al., 2018; Zhou et al., 2018; Radhakrishnan et al., 2019; Butti et al., 2021). Besides secreted factors, key cellular signaling pathways such as TGFB, NFKB, PI3K/AKT have been identified as a central hub in mediating the crosstalk between the cancer cells and CAFs important for tumor metastasis (Erez et al., 2010; Sharon et al., 2015; Ringuette Goulet et al., 2018). Interestingly, recent evidence has also suggested a lesser known juxtacrine-mediated pro-tumorigenic functionality of CAFs (Gaggioli et al., 2007; Labernadie et al., 2017; Chen et al., 2022). Concomitantly, this contact-dependent functionality logically points towards the potential involvement of the Notch signaling pathway, which is a pre-dominant juxtacrine pathway. However, the Notch pathway has been less explored in this aspect, limited to only a few reports (Du et al., 2019; Pelon et al., 2020). Similarly, only few recent studies have focused on the role of Notch signaling in CAF activation (Procopio et al., 2015; Kayamori et al., 2016; Nabet et al., 2017; Meurette and Mehlen, 2018; Song and Zhang, 2018; Pelon et al., 2020; Xue et al., 2021).

Notch signaling is one of the most conserved signaling pathways that plays important developmental roles and has been implicated in tumorigenesis. It is initiated by cell-cell contact between a signal-sending cell expressing Notch ligands on its membrane (Jagged1/2, Delta-like ligand 1/3/4) and a signal-receiving cell expressing Notch receptors (Notch1/2/3/4) (Siebel and Lendahl, 2017; Bray and Gomez-Lamarca, 2018; Sachan et al., 2023). Upon contact, two successive proteolytic cleavages of the receptor lead to activation and nuclear localization of the Notch intracellular domain (NICD). Inside the nucleus, binding of the NICD converts a key transcriptional repressor called CSL (for CBF1, Su(H) and LAG-1) to a transcriptional activator by removal of co-repressors and recruitment of co-activator such as Mastermind like protein (MAML) (Borggrefe and Oswald, 2009; Schwanbeck, 2015; Oswald and Kovall, 2018). This further turns on activation and expression of downstream target genes, notably the Hes and Hey family of proteins (Borggrefe and Oswald, 2009). Notch signaling has been shown to have both tumor suppressive and oncogenic functions, due to its cell and context-dependent pleotropic nature (Lobry et al., 2014; Nowell and Radtke, 2017). However, most of the focus has been on understanding the role of Notch signaling in cancer cells with less focus on its role in the stromal cells, particularly CAFs. Even though recent evidence points towards the role of Notch signaling in the TME (Meurette and Mehlen, 2018; D'Assoro et al., 2022) much remains unexplored. In this review, we have tried to summarize most of the recent reports that have focused on understanding the importance of Notch signaling in the different malignancies and cells of the TME. Our main aim has been to focus on the importance of Notch activation in CAFs as a driver of tumorigenesis and metastasis. We highlight both the oncogenic and tumor suppressive functions of Notch and the clinical trials targeting Notch. We aim to steer the reader’s attention towards the need to drive future research aimed at understanding the role of Notch signaling in CAFs, with the hope of increasing the interest in this field leading to the development of effective therapies targeting the cancer cells and TME simultaneously.

## Notch signaling pathway: simple, yet complicated

The Notch signaling pathway is an evolutionarily conserved juxtacrine pathway that is activated by direct contact between two adjacent cells. The signal sending cell predominantly expresses one of the 5 Notch ligands, namely, Jagged1, Jagged2, Delta-like ligands (DLL1, DLL3, and DLL4), whereas the signal receiving cell predominantly expresses one of the 4 Notch receptors, namely, Notch1,2,3 and 4 (Gozlan and Sprinzak, 2023). The process starts with furin-like convertase mediated S1 cleavage of the Notch polypeptide which converts it into a heterodimer comprising of the notch extracellular, transmembrane, and intracellular domains (NECD-NTM-NICD) (Kovall et al., 2017; Gozlan and Sprinzak, 2023). Contact between a ligand and a receptor leads to force mediated conformational changes in the receptor exposing successive cleavage sites required for signal transduction (Bray and Gomez-Lamarca, 2018; Gozlan and Sprinzak, 2023). This conformational change first exposes the Notch extracellular domain (NECD) for the S2-cleavage that is mediated by the ADAM family of metalloproteinase such as ADAM10, ADAM17. S2 cleavage leads to shedding of Notch ectodomain and what remains is the membrane-tethered truncated part called Notch extracellular truncation (NEXT). This subsequently primes the NEXT for an S3-cleavage mediated by the gamma-secretase complex composed of PSEN1, PSEN2, Nicastrin, PEN2 and APH1 (Bray and Gomez-Lamarca, 2018). The S3-cleavage leads to production of the active Notch intracellular domain (NICD) that localizes to the nucleus in virtue of its nuclear localization sequence (NLS). The Notch signaling pathway is much simpler compared to other cellular signaling pathways owing to non-involvement of secondary messengers and signal transducers. It is the NICD that is the key signal transducer relaying the extracellular signal induction to downstream gene expression changes. Once inside the nucleus, the NICD converts the transcriptional repressor called RBPJ (Recombination signal sequence-binding protein Jk), also known as CSL [for CBF/Su(H)/Lag1] into a transcriptional activator. RBPJ and NICD form a ternary complex called Notch ternary complex or NTC by recruiting the co-activator Mastermind (MAM) that belongs to the Mastermind-like (MAML) family of co-activators involving MAML1, 2 and 3 (Kovall et al., 2017; Bray and Gomez-Lamarca, 2018). Importantly, RBPJ conversion is the key “switch” that decides the shift from a “Notch inactive/OFF” state to a “Notch active/ON” state. This seemingly simple and versatile conversion of RBPJ from a repressed state to an activated state involves important co-effectors that can either form a repressor or an activator complex. Important co-repressors that associate with CBF-1 in the absence of NICD include MINT/SHARP/SPEN, NCoR/SMRT and KyoT2 (Bray and Gomez-Lamarca, 2018; Oswald and Kovall, 2018).

In addition to these well documented proteins, several other key transcriptional and chromatin/epigenetic regulators play equally important roles in the regulatory process. Key chromatin modifiers have recently been identified to co-associate with CBF1 to regulate Notch signaling (Borggrefe and Oswald, 2009; Schwanbeck, 2015; Oswald and Kovall, 2018). Most notable co-repressors include members of the Polycomb repressive complex (PRC2) such as EZH1/2, SUZ12, EED and RING1B; and various histone lysine demethylases (KDMs) such as KDM5A and KDM1 (LSD1) (Borggrefe and Oswald, 2009; Margueron and Reinberg, 2011; Schwanbeck, 2015; Chammas et al., 2020). PCR2 represses Notch target gene expression via di- and tri-methylation of H3K27 residues (Margueron and Reinberg, 2011; Kangsamaksin et al., 2015; Chammas et al., 2020). LSD1 and KDM5A on the other hand repress via demethylation of H3K4me^1/2^ and H3K4me^3^, respectively (Dimitrova et al., 2015). In addition to KDMs, several Histone deacetylases (HDACs) are also known to interact with CSL, namely, SIRT1. SIRT1 deacetylates H4K16 and interacts with PRC2 leading to suppression of gene expression (Li et al., 2020). Other HDACs include HDAC1 that deacetylates H3K9 leading to a repressive mark. HDAC1 also forms a complex with the scaffolding protein CoREST and LSD1 called as the Co-REST complex that also performs suppression of target gene expression via both demethylation of H3K4 and deacetylation of H3K9 (Kalin et al., 2018; Li et al., 2020). In addition to the above epigenetic regulators, certain chromatin remodeling complexes also play critical role in suppressing Notch gene expression such as the NuRD (Nucleosome remodeling and deacetylation corepressor complex) (Schwanbeck, 2015). NuRD often associates with HDAC1 which deacetylates Histone H3 leading to a repressive mark, compaction of chromatin and suppression of gene expression (Clapier and Cairns, 2009; Basta and Rauchman, 2015). On the other end of the spectrum are the co-activators of Notch signaling that are recruited by the CBF-1/NICD/MAML complex to turn up downstream gene expression (Borggrefe and Oswald, 2009; Schwanbeck, 2015). These involve several histone modifying enzymes such as Histone Acetyl Transferases (HATs), p300/CBP, GCN5, and PCAF that can acetylate residues such as H3K9/H3K27 respectively, leading to target gene expression (Borggrefe and Oswald, 2009; Husmann and Gozani, 2019). In addition to these, components of the SWI/SNF family of chromatin remodelers such as SMARC1/2 have been shown to interact with Notch and regulate target gene expression (Schwanbeck, 2015). SWI/SNF recognizes and binds to H3K27ac mark that are typically present in “enhancer” regions and signifies activated transcription (Clapier and Cairns, 2009; Centore et al., 2020). Other notable remodelers include the NuRF (Nucleosome Remodeling Factor) family proteins that function by recognizing activating chromatin mark such as H3K4me^3^ and H4K16ac (Clapier and Cairns, 2009). Other epigenetic and chromatin regulators important in Notch signaling activation includes various histone chaperones such as anti-silencing factor I (ASFI), nucleosome assembly protein I (NAPI) involved in H2A/H2B and H3/H4 remodeling, respectively (Schwanbeck, 2015). Formation of the CBF-1/NICD/MAML trimeric complex along with their several co-activators triggers the switch and can turn on the gene expression downstream of Notch.

The most prominent Notch target genes include members of the hairy enhancer of split (HES) family of bHLH transcriptional repressors, such as Hes1, 5, 7 and members of hairy-related transcription factor (HEY) family, such as Hey1, Hey2 and HeyL (Fischer and Gessler, 2007; Ranganathan et al., 2011). Other notable targets include Cyclin D1 (CCND1), c-Myc, Notch receptors and ligands, etc. (Ranganathan et al., 2011; Zhou et al., 2022). The downstream effects of Notch activation include regulation of cell fate differentiation, cellular proliferation/apoptosis, stem cell development, etc. (Andersson et al., 2011; Siebel and Lendahl, 2017; Gozlan and Sprinzak, 2023; Sachan et al., 2023). Upon activation of downstream targets, homeostasis needs to be maintained, which involves turning off the signal. This is crucially mediated by degradation of the NICD, which is triggered by its phosphorylation by CDK8 kinase, in turn making the phosphorylated NICD a substrate for the E3 ubiquitin ligase, Sel10/FBW7 leading to its proteasomal degradation (Bray, 2016). The degraded NICD is no longer available to maintain the NTC leading to its dissociation. Other areas of Notch signaling regulation involve Notch ligand endocytosis/trafficking mediated by E3 ubiquitin ligases Neutralized 1,2, Mindbomb, Skeletrophin and receptor endocytosis/trafficking by Deltex 1-4, Nedd4 and AIP4 (Bray, 2016). Additionally, post-translational modifications of Notch receptors such as O-fucosylation and O-glucosylation mediated by Pofut-1 and Fringe family of proteins, are also found to be important for their functionality and proper ligand binding (Kopan and Ilagan, 2009; Bray, 2016; Kovall et al., 2017).

## Notch signaling in metastasis

Cancer metastasis comprises of a well-coordinated action of various factors and signaling pathways that harmonize to drive migration and invasion of primary cancer cells to eventually form secondary metastatic tumors in different organs. This involves a multistep process that is primarily initiated by cells undergoing “Epithelial to Mesenchymal transition (EMT).” EMT is a well-studied developmental process that involves loss of epithelial characteristics in the form of loss of cellular adhesion and attachment and eventual gain of mesenchymal properties exhibiting spindle shapes and increased motility of cells (Dongre and Weinberg, 2019). The process of EMT is characterized by loss of epithelial markers such as E-cadherin, EpCAM and gain of mesenchymal markers such as N-cadherin and vimentin (Dongre and Weinberg, 2019). EMT is an important hallmark of cancer and metastasis and is driven by both intrinsic and extrinsic cellular and environmental cues that have been an important focus of research. Cellular signaling pathways have been shown to play a paramount role in this process and have been a hot target for therapies for metastasis. An array of transcription factors play an important role in regulating the process of EMT, involving upregulation of mesenchymal transcription factors such as, Slug, Snail, Zeb and Twist1 (Kalluri and Weinberg, 2009; Brabletz et al., 2018; Dongre and Weinberg, 2019).

One of the earliest roles of Notch signaling in EMT was reported by Leong et al. (2007) in breast cancer mouse model. They demonstrated that overexpression of N1ICD and N4ICD led to decreased expression of E-cadherin in normal breast epithelial cells, which was mediated by induction of the transcription factor Slug, but did not affect either Snail or Twist1, enabling the cells to gain a mesenchymal-like phenotype (Leong et al., 2007). The Slug promoter contained two CSL binding sites and an EMSA-based assay further confirmed Slug as a directly bound target of the Notch/CSL complex. Co-culturing experiments showed that induction of Slug was mediated by a Jagged1-Notch juxtacrine loop (Leong et al., 2007). Inhibition of Jagged1-Notch4 interaction, by using a soluble Notch4 receptor, decreased Slug expression and led to re-expression of E-cadherin in mouse xenografted tumors with further inhibition of metastatic burden. Interestingly, the re-expression of E-cadherin was via reduction in E-cadherin promoter DNA methylation and the Notch mediated Slug expression was important in protecting the cells from anoikis, further helping in metastasis (Leong et al., 2007). A similar study corroborating the above findings was reported by Shao S. et al. (2015). These studies demonstrated a significant finding connecting Notch induction and EMT via Slug upregulation with a potential for therapy. The crosstalk linking Hypoxia to Notch signaling has been demonstrated to be necessary for the enrichment and sustenance of CSCs and mediation of chemoresistance (Seo et al., 2016). This crosstalk was explored further to understand its importance in cancer metastasis and couple of important works were reported (Sahlgren et al., 2008; Chen et al., 2010; Xing et al., 2011). Sahlgren et al. (2008) reported the induction of Notch signaling in cells when cultured under hypoxic condition vs. normoxic condition that could be abrogated by using GSI. Further, hypoxia exhibited dual ability in inducing Notch ligand, DLL1 and cooperated with already active Notch signaling to further potentiate the output (Sahlgren et al., 2008). Interestingly, by using Notch^high^ and Notch^low^ expressing cell lines, it was demonstrated that hypoxia-mediated induction of EMT required an already active baseline Notch signaling and cells having low or blocked-Notch signaling displayed no effect towards hypoxia-mediated EMT (Sahlgren et al., 2008). Co-IP and ChIP experiments revealed the presence of HIF1α-N1ICD-MAML1 transcriptional complex that was responsible for EMT induction via Snail1. The presence of CSL binding site in the Snail1 promoter was critical for N1ICD-mediated Snail1 expression as deletion of this binding site led to loss of Snail1 promoter activation in the presence of N1ICD (Sahlgren et al., 2008). Interestingly, Snail1 was revealed as a direct target of N1ICD and HIF1α could only bind at the Snail1 promoter under combined conditions of hypoxia and Notch-induction. Besides this primary mechanism, a secondary effect of N1ICD via potentiation of a HIF1α-LOX-Snail1 axis was also shown to be synergistic for EMT (Sahlgren et al., 2008) revealing two potential therapeutic targets for metastasis. Interestingly, findings from Leong et al. (2007) revealed the importance of Slug but not Snail for Notch-mediated EMT induction in breast cancer whereas, Sahlgren et al. (2008) demonstrated that it was Snail1 that was responsible for EMT. This difference could be explained due to the cancer- and context-dependent pleotropicity known to be exhibited by Notch signaling and requires more research. The existence of a synergistic cooperation between HIF1α or HIF2α and MAML1, hypothesized to be important towards NICD3 mediated invasion of breast cancer cells, was also reported (Chen et al., 2010).

An interesting but different take on the crosstalk between hypoxia and Notch in cancer metastasis was reported by Xing et al. (2011), this time looking at the role of Notch ligand Jag2 for the same. IHC in breast cancer patient samples revealed the upregulation of NICD1 and Jag2 in the invasive front rather than within the core of the tumor, suggesting Jag2 mediated Notch1 activation playing a role in breast cancer invasion and metastasis. Co-staining of NICD1 with hypoxic markers CA9 and GLUT1 was also prevalent in the invasive front indicating hypoxia-Jag2-Notch1 as a potential mechanistic axis. Interestingly, coculturing of Jag2-expressing bone marrow stromal cells with breast cancer cells led to increase in Notch reporter activity only under hypoxic condition (Xing et al., 2011), indicating the role of Jag2-expressing hypoxic stroma in cancer cell Notch activation. Mechanistically, the hypoxia-Jag2-Notch1 axis between breast cancer cells and bone marrow stromal cells was shown to be important for metastasis and for the enrichment of BCSCs (Xing et al., 2011). Understanding the activation of Jag2 by hypoxia will provide relevant avenues for therapies targeting Jag2 in both cancer cells and stroma. The authors reported a lack of any HIF1α binding site in Jag2 promoter, which further makes it more interesting and highlights the need for understanding the mechanisms leading to hypoxia mediated Jag2 upregulation. One probable explanation could be that Jag2 is bound and regulated by HIF2α instead of HIF1α, as it has been well established that HIF1α and HIF2α can be part of larger distinct transcriptional complexes, demonstrate HIF1α–HIF2α switching, and have gene-specific regulatory roles (Keith et al., 2011).

A more recent and comprehensive study elucidating the role of Notch ligand DLL1 in luminal breast cancer progression and metastasis was reporter by Kumar et al. (2019). DLL^high^ ER^+^ breast tumor patient tissues displayed significantly poor OS and distant metastasis-free survival (DMFS) compared to normal breast tissues. Knockdown of DLL1 led to decrease in breast tumor volumes and reduced lung metastases, whereas DLL1 overexpression increased the same *in vivo* (Kumar et al., 2019). Further investigation revealed the possible involvement of DLL1 in mediating Erα^+^ luminal breast cancer progression by induction of tumor cell proliferation and angiogenesis. The tumor promoting function of DLL1 in ERα^+^ luminal breast tumors was initiated by estrogen-mediated inhibition of DLL1 ubiquitination and degradation, leading to increase in DLL1 protein stability (Kumar et al., 2019). This study has a significant impact towards sub-type specific targeted therapeutic opportunities for patients with breast cancer. One notable aspect of this study was the fact that DLL1 in Erα^−^ breast cancer cells apparently seemed to have a tumor suppressive function in contrary to the oncogenic function of DLL1 in Erα^+^ breast tumors. This is not surprising as Notch signaling is known to function both as an oncogene and tumor suppressor depending on the tumor type. Interestingly, Notch signaling in HER2^-^ breast CTCs has been reported to mediate chemoresistance (Jordan et al., 2016) while, a contradicting study has signified the role of Notch1 in mediating CSC survival in HER2^+^ breast cancer (Baker et al., 2018), further pointing towards the complexity and pleotropicity of Notch signaling in cancer.

Liu et al. (2021) have delineated the importance of NICD1 in mediating NSCLC metastasis and stemness. The authors identified differential expression of the DNA replicator factor RFC4, when NICD1 was overexpressed in NSCLC cells. The effect of NICD1-mediated RFC4 functioning was shown to increase proliferation, stemness and metastatic abilities in these cells both *in vitro* and *in vivo* (Liu et al., 2021). RFC4 was found to contain binding site of Notch transcription factor, CSL and was shown to be a direct transcriptional target of Notch signaling. The authors identified a positive feedback loop that regulates the Notch1-RFC4 axis, where RFC4 amplifications and Notch1-mediated RFC4 upregulation can in turn prevent the degradation of NICD1 by CDK8/FBXW7 further leading to NSCLC metastasis (Liu et al., 2021). This was a significant finding paving the way for GSI- and NICD1-RFC4 inhibition mediated dual targeting of NSCLC metastasis. However, it remains to be determined if RFC4’s protective role is specific for NICD1, or if it also displays a similar protection to other NICDs from getting degraded. Notch signaling has also been implicated to have pro-metastatic functions in high grade serous ovarian cancer (HGSOC) (Huang et al., 2019). By analyzing tissue microarrays from 221 ovarian cancer patients, Huang et al. (2019) identified significant correlation between non-canonical Notch ligand, DLK1 with patient tumor stages and lymph node metastasis. Interestingly, DLK1 upregulation was correlated with poor OS and PFS, along with negative correlation with the EMT marker, E-cadherin, only in patients with HGSOC and not any other subtypes. Overexpression of DLK1 was shown to increase migration, invasion and clonogenicity of HGSOC cells while DLK1 knockdown had reverse effects, indicating its role in metastasis. In mice xenograft models, DLK1 overexpression exerted its effects by increasing proliferation and neovascularization of the tumors, leading to increased tumor volume. This effect of DLK1 was shown to be mediated by Notch1 and not Notch3, as Notch1 neutralizing antibody had more potent effect in curbing the invasiveness of HGSOC cell lines compared to that of Notch3 (Huang et al., 2019). Importantly, DLK1 is a non-canonical Notch ligand that seems to activate Notch1 downstream in this context. Accordingly, it would be interesting to decipher the crosstalk regulating this process and the roles of canonical Notch ligands in the same.

Although there has been less focus on the role of Notch signaling in ovarian cancer metastasis, there is some evidence indicating the potential role of Notch in the process (Gupta et al., 2013; Han et al., 2020). Overexpression of active NICD3 in epithelial ovarian cancer cells was shown to induce mesenchymal markers, αSMA, Snail and Slug, while decreasing expression of E-cadherin, concomitantly leading to a mesenchymal phenotype (Gupta et al., 2013). This NICD3 induced EMT activation was thought as a potential mechanism of carboplatin-resistance in these cells. Notch3 induced ERK phosphorylation was shown to mediate cisplatin-resistance in ovarian cancer cells (Gupta et al., 2013). The microRNA, miR-1271-5p was reported to regulate ovarian cancer progression and invasion via the Notch signaling pathway *in vitro* (Han et al., 2020). The authors identified a tumor suppressive role of miR-1271-5p that directly targeted TIAM1. Importantly, miR-1271-5p level was observed to be downregulated in ovarian cancer which in turn increased TIAM1 expression, leading to increased clonogenicity and invasive ability of these ovarian cancer cells. Increased TIAM1 was shown potentially to regulate the expression of Notch1 pathway genes such as Notch1 itself, Hes1, Cyclin D1 and negative regulator NUMB (Han et al., 2020). This unique miR-1271-5p-TIAM1-Notch1 axis in ovarian cancer cells provides a novel mechanism of targeting multiple players alone or in combination. One interesting perspective of Notch mediated metastasis in hepatocellular carcinoma (HCC) was reported by Jin et al. (2019). By modulating the expression levels of mitochondrial Ca^2+^ uniporter regulator, MCUR1 they showed that MCUR1 played important role in ROS mediated EMT in HCC both *in vitro* and *in vivo*. The increased ROS production played a crucial role in regulating the nuclear localization of Nrf2 which further activated expression of Notch1 (Jin et al., 2019). This Nrf2 mediated Notch1 induction led to EMT in HCC cells by increase in Snail, N-Cadherin and Vimentin, while decreasing expression of ZO-1 and E-cadherin. This effect could be reversed by addition of GSI in cells overexpressing MCUR1, indicating opportunities for therapy. This study provides an interesting approach towards targeting metastatic HCC cells via dual inhibition of mitochondrial Ca^2+^ influx and Notch activation. Importantly, the authors have shown that silencing of Nrf2 led to inhibition of NICD1 activation, further pointing towards understanding of the molecular mechanism behind Nrf2 mediated Notch1 upregulation (Jin et al., 2019).

Since NICD’s nuclear translocation is a crucial step towards Notch downstream gene activation, it would be important and interesting to see if Nrf2 interacts with the NICD/CSL complex in this process. We have previously discussed the highly pleotropic and context-specific role of Notch signaling in cancer, which is also true for the regulation of cancer metastasis. Accordingly, Zou et al. (2019) uniquely identified an anti-metastatic role of Notch signaling, specifically Notch2 in nasopharyngeal carcinoma (NPC). Notch2 expression was shown to be downregulated in NPC metastatic patient biopsy samples compared to primary tumors. Notch2 and NICD2 were also decreased in poorly differentiated NPC samples (higher grade) and correlated with significantly poorer OS, all of which indicated a potential metastasis-suppressive role of Notch2 (Zou et al., 2019). The silencing of Notch2 showed increased invasion and migration of NPC cells *in vitro*, while also increasing liver and lung metastases *in vivo*. Correspondingly, the reverse effect was produced by the overexpression of Notch2. Interestingly, metastasis suppressive effect of Notch2 was shown to be mediated by suppression of TRAF6/Akt/P70S6K/mTOR signaling axis (Zou et al., 2019). This study indicates the potential of multiple combinatorial therapeutic approaches. However, the underlying mechanism mediating the effect of the interaction between TRAF6 and Notch2 needs to be understood in detail for design of specific inhibitory molecules. The numerous upstream activators of Notch signaling and its downstream mediators are summarized in Table 1.

**TABLE 1:** Summary of Notch activators and downstream signaling pathways.

Notch signaling in metastasis			
Cancer type	Notch pathway components	Associated non-Notch components	References
Breast	NOTCH1, NOTCH3, NOTCH4, JAGGED1, JAGGED2, DLL1, MAML1	E-Cadherin, Slug, Snail, LOX, HIF1α, HIF2α	Leong et al. (2007), Sahlgren et al. (2008), Chen et al. (2010), Xing et al. (2011), Shao et al. (2015b), Kumar et al. (2019)
Lung	NOTCH1, CSL, CDK8, FBXW7	RFC4	Liu et al. (2021)
Ovarian	NOTCH1, NOTCH3, DLL1, DLK1, HES1, CCND1, NUMB, MAML1	E-Cadherin, Snail, Slug, αSMA, p-Erk, miR-1271-5p, TIAM1, HIF1α, LOX	Sahlgren et al. (2008), Gupta et al. (2013), Huang et al. (2019), Han et al. (2020)
Hepatocellular	NOTCH1	MCUR1, NRF2, Snail, N-Cadherin, Vimentin, ZO-1, E-cadherin	Jin et al. (2019)
Nasopharyngeal	NOTCH2	TRAF6, AKT, P70S6K, mTOR	Zou et al. (2019)

Notch signaling in TME			
Cell type	Notch pathway components	Associated non-Notch components	References
Endothelial cells	NOTCH1, NOTCH2, NOTCH3, DLL4, JAGGED1	VEGF, sVEGFR-1	Benedito et al. (2009), Blanco and Gerhardt (2013), Liu et al. (2014), Kangsamaksin et al. (2015), Kuhnert et al. (2015), Lugano et al. (2020), Akil et al. (2021), Luo et al. (2022)
Immune cells	NOTCH1, NOTCH2, JAGGED1, JAGGED2, DLL4, HES1, RBPj, FBXW7	miR-125a, FIH1, IRF4, SIRPα, Arginase I, NFKβ, iNOS, MCT2, CCL2, CSF1, c-Jun, CD68, COX2	DeHart et al. (2005), Kijima et al. (2008), Wang et al. (2010), Zhao et al. (2016), Sierra et al. (2017), Lin et al. (2018), Huang et al. (2021), Zhao et al. (2022)
Cancer associated fibroblasts (CAFs)	NOTCH1, NOTCH2, NOTCH3, NOTCH4, CSL, HES1, HEY1, HEY2 HES4, c-MYC	αSMA, PDGFRα, FGF7, IGF2, MMP3, 9, 13, c-Jun, Fos, CDKN2B, 2A, 1A, p53, PDCD4, WISP1, TGFβ, CD271, Nestin, RN7SL1, C/EBPB, PDGFRβ, Collagen I, Fibronectin	Hu et al. (2012), Procopio et al. (2015), Hoare et al. (2016), Jo et al. (2016), Nabet et al. (2017), Song and Zhang (2018), Du et al. (2019), Strell et al. (2019), Shao et al. (2021), Xue et al. (2021), Kim et al. (2022), Luo et al. (2022)

## Cancer associated fibroblasts (CAFs)—key component of the TME

**FIGURE 1:**
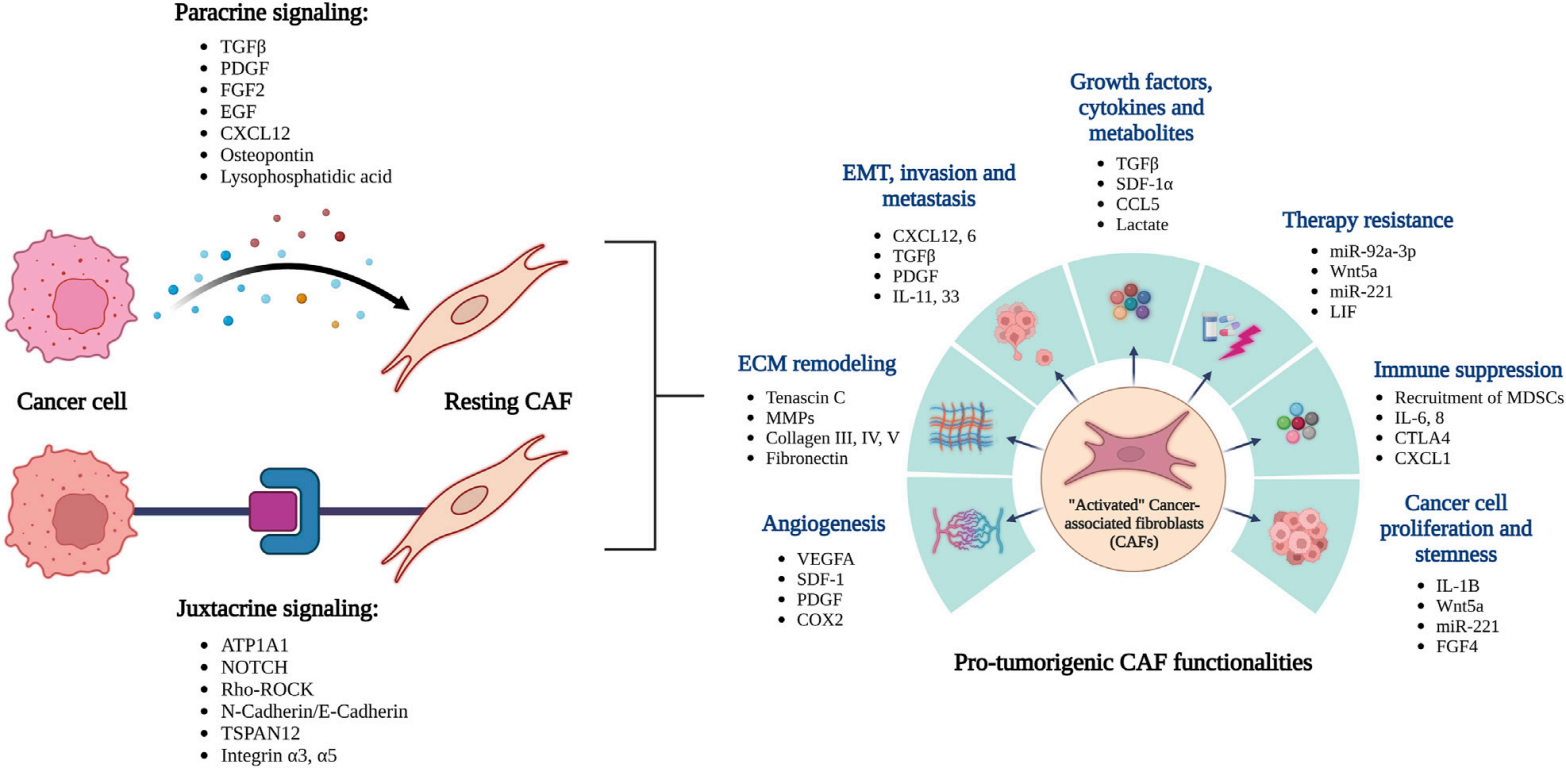
Overview of the reciprocal interactions between cancer cells and CAFs leading to their activation. The activated CAFs can promote tumor growth and spread through a variety of functions.

In addition to the cancer cells, the tumor microenvironment (TME) largely comprises of a collection of stromal cells including endothelial cells, cancer associated fibroblasts (CAFs), a repertoire of immune cells, adipocytes, various acellular components like secreted factors and ECMs. This milieu of stromal cells and other stromal components create a highly favorable “niche/soil” to sustain the new metastases. A large pool of data has recently focused on understanding how this favorable niche is built by the invading cancer cells, which eventually helps establish metastases (Figure 1). Results have largely indicated that this process is predominantly governed by the highly dynamic and productive crosstalks between the metastasizing cancer cells and stromal cells of the new TME. Among all the stromal cells of the TME, a heterogeneous group of fibroblasts named as “Cancer Associated Fibroblasts or CAFs,” are usually the most prevalent (Sahai et al., 2020; Chen et al., 2021; Wang et al., 2021). CAFs are defined largely as fibroblasts present in tumors in close proximity to the cancer cells. CAFs are highly heterogenous and can be classified into various sub-types based on their distinguishing surface markers, secreted factors, origin, and unique functionalities towards cancer development (Gascard and Tlsty, 2016; Sahai et al., 2020; Wang et al., 2021). However, CAFs can broadly be grouped into two large types based on whether they are tumor-restraining (rCAFs) or tumor-promoting (pCAFs) (Wang et al., 2021). In addition to that, researchers have also classified CAF subtypes based on other criteria such as phenotypic characteristics, surface markers, etc. (Lavie et al., 2022; Caligiuri and Tuveson, 2023; Chhabra and Weeraratna, 2023) which calls for the need to better understand their heterogeneity and plasticity.

A large pool of data has been reported on CAF functionalities in respect to tumor progression, both as tumor-restrainers and/or tumor-promoters. Rhim et al. (2014), observed that genetic deletion of the Shh ligand in the epithelial tumor cells led to development of more aggressive PDAC tumors in mice, while exhibiting reduced stroma (fibroblasts and macrophages), indicating potential tumor-restraining properties of the stroma (Rhim et al., 2014). Interestingly, the Shh deletion led to increased vasculature in these tumors even though the tumor sizes were smaller. Further studies led them to propose that the PDAC epithelial cells played some role in activating certain stromal cell populations via the Hedgehog signaling which happen to display some tumor-restraining properties (Rhim et al., 2014). This was a seminal finding involving the role of stromal fibroblasts as tumor-restrainers. A similar study was reported by Ozdemir et al. (2014) at the same time. By using an ingenious approach, the authors performed deletion of proliferating αSMA^+^ myofibroblasts at different stages of PDAC development in mice. These αSMA^+^ myofibroblast depleted mice developed more aggressive tumors and displayed shorter OS (Ozdemir et al., 2014). Interestingly, this depletion also resulted in decreased type I collagen and ECM stiffness while, significant remodeling of the same. This depletion also entailed decreased tumor vasculature, indicating that observations from Rhim et al. (2014)’s study might be driven by alternative mechanisms. The most striking outcome of αSMA^+^ myofibroblast depletion came forth in the development of an immuno-suppressive microenvironment via decrease in T_eff_/T_reg_ ratio and elevated CTLA4 expression (Ozdemir et al., 2014). This study very specifically denoted the tumor-restraining role of αSMA^+^ myofibroblasts in PDAC that was potentially mediated via modulation of the immune microenvironment, indicating the potential for combinatorial therapeutic approaches. Importantly, the authors also identified a panel of 4393-differentially expressed genes in the early αSMA^+^ myofibroblast depleted mice vs. control (Ozdemir et al., 2014). Analyzing this data would potentially provide a landscape of important cellular events and factors that are important in generating and/or maintaining these tumor-restraining CAFs which can be harnessed for therapy. A more recent study by Mizutani et al. (2019) aimed at identifying specific marker-expressing tumor-restraining CAFs in PDAC. The authors identified a specific sub-population of rCAFs (αSMA^low^PDGFRa^+^Gli^+^) that exhibited Meflin as a unique marker in PDAC (Mizutani et al., 2019). Interestingly, this sub-population of Meflin^+^ rCAFs were speculated to be a potential source of αSMA^+^CAFs during PDAC progression. Further, Meflin-KO mice exhibited PDAC tumors that were poorly differentiated that aligns with previous findings (Ozdemir et al., 2014; Rhim et al., 2014), regarding undifferentiated PDAC tumors in the absence of rCAFs. Therapeutically, Meflin can be potentially thought to be used to restrain PDAC progression by enhancing populations of rCAFs since the authors have demonstrated the same (Mizutani et al., 2019). Further details about tumor-restraining functions of CAFs are beyond the scope of this paper and can be found in a detailed review by Wang et al. (2021).

Besides the tumor-restraining function, CAFs have been more widely reported for their tumor-promoting functions, including promoting metastasis. Accordingly, CAFs have been shown to aid in tumor progression and metastasis via a repertoire of mechanisms, including, secretion of pro-tumorigenic growth factors and cytokines, ECM remodeling via secretion of type III/IV/V collagens, laminins, and fibronectins; immunosuppression, induction of EMT in cancer cells, changes in the metabolic landscape, among others (Gascard and Tlsty, 2016; Kalluri, 2016; Sahai et al., 2020). Therefore, CAFs have been shown to be highly versatile in their pro-metastatic functionalities. They can promote metastasis either via the secretion of pro-metastatic factors acting in a paracrine fashion on cancer cells or via direct-contact mediated enhancement of invasive/metastatic ability of cancer cells via a juxtacrine fashion (Kalluri, 2016; Sahai et al., 2020).

## Secretory and paracrine functions of pro-metastatic CAFs

The pro-metastatic functions of CAFs mediated in a paracrine fashion have been well documented and reported by various works (Orimo et al., 2005; O’Connell et al., 2011; Calon et al., 2012; Ershaid et al., 2019; Hu J. L. et al., 2019; Pelon et al., 2020; Zhou et al., 2020). O’Connell et al. (2011) identified the enrichment of S100A4^+^ stromal cells in the TME of breast cancer metastases compared to primary TME in both mice model and patient samples. Further characterization identified these S100A4^+^ stromal cells predominantly as fibroblasts, whose selective genetic ablation led to significant reduction of metastatic colonization and increased apoptosis in cancer cells. These S100A4^+^ fibroblasts were shown to mediate metastatic colonization via secretion of various ECM proteins such as Tenascin-C and by the induction of angiogenesis via secretion of VEGF-A (O’Connell et al., 2011). This subset of stromal fibroblasts thus, have a dual role in providing a supportive metastatic “niche/soil” for the invading cancer cells. Targeting of these cells is expected to have greater effect in reducing metastatic colonization due to their dual functions. Orimo et al. (2005) created a xenograft mouse model where they mixed different types of patient-derived fibroblasts with cancer cells and engrafted them into mice to look at their effect on tumor formation *in vivo*. CAFs displayed the highest ability to promote tumor formation in mice as compared to counterpart and normal fibroblasts. This pointed towards a distinct characteristic of CAFs that were isolated from carcinoma tumors in meditating tumorigenic properties. These CAFs were also shown to possess characteristics of “activated” or myofibroblasts, and a positive correlation was observed between the contractile abilities and tumorigenic properties of these CAFs (Orimo et al., 2005). Further mechanistic studies revealed that these tumor-stromal CAFs secreted SDF-1, which was important for tumor growth and angiogenesis. The CAF-derived SDF-1 could recruit EPCs via an endocrine mechanism and at the same time also induced proliferation of CXCR4-expressing cancer cells in a paracrine manner (Orimo et al., 2005). This dual role of CAF-derived SDF-1 indicates two potential axes of therapeutic opportunities. Calon et al. (2012) reported that FAP^+^CAFs isolated from CRC patients demonstrated significant upregulation of TGFβ responsive gene signatures (TGFBRS) that positively correlated with increased disease relapse after therapy. By using different engineered mouse xenograft models, they further demonstrated that TGFβ signaling in the stromal cells was crucial for the formation of metastases, but not for tumor initiation. Further molecular studies revealed a tumor-stromal crosstalk where TGFβ secreted by CRC cells triggered secretion of IL-11 in CAFs, which further enhanced CRC metastases, in turn by activating STAT3 signaling in the CRC cells (Calon et al., 2012). Importantly, pharmacological inhibition of TGFβ signaling in the stromal cells and/or genetic inhibition of STAT3 signaling in the cancer cells ablated this crosstalk and significantly reduced CRC metastases, further pointing towards the importance of CAF-secreted cytokines for the establishment of metastasis (Calon et al., 2012).

By using a panel of five different CAF markers, Pelon et al. (2020) isolated four different subsets of CAFs from breast cancer patients with primary tumors and lymph node metastases. Interestingly, the metastasized lymph node tumors comprised of enriched CAF subsets S1 and S4, where subsets S1 and S4 displayed differential mechanisms in promoting metastasis (Pelon et al., 2020). CAF subset S1 was identified to be unique in its ability to drive the migration and invasion of the cancer cells by the initiation of an EMT program (Pelon et al., 2020). This CAF-S1-induced EMT program was driven via the secretion of pro-metastatic chemokine CXCL12 and the cytokine TGFβ, which could be ablated by either the genetic knockdown of CXCL12 or the pharmacological inhibition of TGFβ, further pointing towards the potential of CAF-targeted anti-metastatic therapies (Pelon et al., 2020). Zhou et al. (2020) identified an important cancer cell-CAF crosstalk that was key for the metastatic ability of gastric cancer (GC) cells. By analyzing GC patient samples in comparison to corresponding non-cancerous tissues, they demonstrated the selective overexpression of the interleukin IL-33 in CAFs and that of its receptor, ST2L in the cancer cells in these tissues (Zhou et al., 2020). This expression of IL-33 further correlated with activated CAF markers αSMA and FAP, while ST2L expression correspondingly correlated with the epithelial marker cytokeratin in the cancer cells. Clinically, these overexpression profiles are associated with higher local invasion and tumor-node metastasis (TNM) stages, while also being correlated with poor overall patient survival (Zhou et al., 2020) thus, indicating importance in GC metastasis. Further molecular studies led to the identification of a two-way crosstalk that was key in driving this overexpression profiles. The authors identified that GC cell-secreted TNFα was responsible for driving the expression of IL-33 in CAFs via a TNFR2/NFKβ/IRF-1 axis (Zhou et al., 2020). Interestingly, this CAF-released IL-33 in turn induced EMT in GC cells via the activation of ERK1/2-SP1-ZEB2 axis, thus providing a positive feedback loop. Expectedly, inhibition of this crosstalk led to abrogation of the migratory and invasive ability of the cancer cells. Importantly, prior knockdown of either IL-33 in CAFs or ST2L in the cancer cells significantly reduced peritoneal metastatic tumor nodules *in vivo*, further adding to the therapeutic potential of targeting key players of this crosstalk (Zhou et al., 2020).

Ershaid et al. (2019) demonstrated the paracrine role of CAFs in breast cancer metastasis from a different perspective, this time in respect to the inflammasome complex. The authors demonstrated significant upregulation of the NLRP3 inflammasome pathway components in fibroblasts isolated from mice mammary carcinoma as compared to fibroblasts isolated from normal mammary tissues (Ershaid et al., 2019). This upregulation was also observed in human breast carcinoma samples that further correlated with increased disease progression. This activation of the NLRP3 inflammasome in the CAFs could be triggered by the use of various DAMPs such as, H_2_O_2_, tumor-derived necrotic fluid, etc., demonstrating the ability of CAFs to sense DAMPs, previously not implicated (Ershaid et al., 2019). Further molecular studies interestingly demonstrated that NRLP3-mediated secretion of IL1-b by the CAFs was key for mediating tumor growth and metastasis *in vivo* since, co-injection of (nrlp3^−/−^) tumor cells with either (nrlp3^−/−^) or (il1b^−/−^) led to reduced lung metastasis (Ershaid et al., 2019). Notably, the authors identified dual mechanisms via which the NRLP3-mediated IL1-b secretion by CAFs promoted breast cancer metastasis: by the recruitment of immunosuppressive MDSCs, and by inducing expression of MMPs in the cancer cells further leading to increased extravasation and invasion (Ershaid et al., 2019). Hu J. L. et al. (2019) demonstrated the role of CAF-secreted exosomes in promoting metastasis and chemoresistance in CRC. They demonstrated that CAF-secreted exosomes carried and transferred the microRNA miR-92a-3p in the CRC cells. The increased expression of this miRNA further led to decrease in its downstream targets FBXW7 and MOAB1, which play roles in the beta-catenin and mitochondrial apoptosis pathways, respectively (Hu J. L. et al., 2019). miR-92a-3p-mediated suppression of both pathways led to increase in CRC stemness, EMT, while decreasing apoptosis, collectively contributing to increased metastasis and drug-resistance (Hu J. L. et al., 2019). Importantly, increased expression of exosomal miR-92a-3p was detected in the serum of metastatic CRC patients pointing towards potential use of this miRNA as a diagnostic marker, in addition to being a therapeutic target for CRC metastasis (Hu J. L. et al., 2019).

## Juxtacrine functions of pro-metastatic CAFs

Gaggioli et al. (2007) were one of the earliest ones to show that in addition to undergoing motile characteristics, SCC cells require force- and protease-mediated remodeling of the ECM by stromal CAFs for successful invasion. Interestingly, this invasive cascade of SCC cells was mediated by direct contact-mediated guided track created by leader CAFs that depended on cell-adhesion and cytoskeleton remodeling pathways involving integrin α3, α5 and Rho-ROCK, respectively (Gaggioli et al., 2007). This work put forward a new model describing the importance of physical matrix remodeling by leader CAFs for cancer cell invasion and brings forward therapeutic opportunities targeting cell-adhesion/contact between cancer cells and CAFs. This work laid out the idea that CAFs can mediate metastasis not only by the traditionally known paracrine secretion of factors, but also by contact-mediated invasion of cancer cells via creation of a guidance mediated track/path through the ECM. Chen et al. (2022) interestingly demonstrated a differential enrichment of αSMA^+ve^ fibroblasts in PDAC patient tumor specimens; only fibroblasts that were within direct tumoral periphery were positive for αSMA staining as compared to global distribution of vimentin^+ve^ fibroblasts, indicative of contact mediated activation of fibroblasts. Clinically, PDAC patients with high juxta tumoral αSMA^+ve^ fibroblasts demonstrated poor PFS, and importantly, αSMA^+ve^ fibroblasts were also found to be in association with CK19^+^ cancer cells in isolated circulating tumor micro-emboli of PDAC patients (Chen et al., 2022). *In vitro* coculture experiments also demonstrated that direct contact of cancer cells with fibroblasts increased the number of tumor spheroids along with increased tumor cell proliferation and invasion. Mechanistically, Activin A was identified as a key secreted cytokine by the fibroblasts that led to EMT of the cancer cells that could be abrogated by usage of anti-activin A antibody or follistatin both *in vitro* and *in vivo* (Chen et al., 2022). Further molecular insights revealed that homophilic ATP1A1 binding between cancer cells and fibroblasts induced activin A secretion by fibroblasts via the intracellular Ca^2+^-mediated NF-kβ signaling axis. Additionally, activin A also acted in an autocrine manner to induced myofibroblastic differentiation of fibroblasts (Chen et al., 2022). Thus, this cancer-fibroblast crosstalk mediated signaling axis demonstrated dual functionality in PDAC progression and invasion presenting an attractive therapeutic opportunity.

The role of p53 downregulation in CAFs for the contact mediated induction of cancer cell proliferation and invasion was identified in lung cancer (Otomo et al., 2014). Otomo et al. (2014) identified a regulatory loop involving induction of the membrane protein TSPAN12 in p53 downregulated CAFs. Interestingly, cancer cell mediated downregulation of p53 was observed only in CAFs that were in direct contact with the cancer cells, and these CAFs exhibited increased TSPAN12 and enhanced cancer invasion via β-catenin-CXCL6 secretion. This was clinically relevant since expression of αSMA was negatively correlated with p53 expression in stromal tissues from patients (Otomo et al., 2014). Besides, the already mentioned role of homophilic membrane protein interactions between cancer cells and CAFs for metastasis (Chen et al., 2022), importance of heterophilic membrane protein interactions has also been demonstrated (Labernadie et al., 2017). By using squamous cell carcinoma patient-derived CAFs and cancer cells, Labernadie et al. (2017) demonstrated that heterophilic E-cadherin/N-cadherin interactions between cancer cell and CAFs, were crucial for SCC invasion. By exhibition of pulling forces via E-cad/N-cad junctions with cancer cells, CAFs could guide the invasion of cancer cells both in 2D and 3D. Interestingly, rapid and colocalization of E-cad and N-cad was observed in cancer cell-CAF junctions which could be abrogated by mutation of E-cad in the cancer cells (Labernadie et al., 2017). Importantly, KO of E-cad in cancer cells or KD of N-cad in CAFs led to ablation of CAF-mediated invasion of cancer cells. N-cad KD CAFs were unable to pull wild-type cancer cells and similarly, E-cad KO cancer cells were unable to follow wild-type CAFs, demonstrating the requirement of this heterophilic interaction for SCC invasion (Labernadie et al., 2017).

Recently, contact-mediated interaction between cancer cells and CAFs has also been shown to be key for driving aggressiveness of cancer. Gao et al. (2019) intriguingly demonstrated the critical role of CAFs for the formation of contact-mediated heterotypic spheroids with ascitic tumor cells for the aggressiveness observed in HGSOC. By comparing ascites fluid collected from HGSOC vs. LGSOC patients, the authors observed increased ability of HGSOC-derived ascitic cancer cells to form aggregates due to an enrichmen of CAFs. Intriguingly, intraperitoneal injection of a mixture of CAFs and ascites ovarian cancer cells in mice, led to instantaneous formation and adhesion of heterospheroids to the omentum, development of tumor nodules and corresponding appearance of the heterospheroids into the ascites, all of which are key for metastasis (Gao et al., 2019). By profiling tumor cells capable of forming heterospheroids with CAFs, the authors demonstrated integrin alpha 5 (ITGA5), an important protein for cellular adhesion, to be highly upregulated pointing towards the importance of cancer cell-CAF contact for metastasis. The authors further demonstrated the formation of heterospheroids comprised of a CAF core with attached ascitic tumor cells which were key for peritoneal spread and adhesion to remote metastatic sites (Gao et al., 2019). Importantly, by selectively using a small molecule inhibitor of the PDGF signaling, called imatinib, to target CAFs, the authors observed disruption of the CAF core which further prevented heterospheroid formation, reduced peritoneal adhesion while also increasing apoptosis of the cancer cells (Gao et al., 2019). Strikingly, pre-treatment of CAFs with imatinib displayed reduced peritoneal metastasis, tumor stroma and improved survival of mice while not affecting the primary tumors (Gao et al., 2019). This study critically points towards the relevance and importance of selectively targeting CAFs to inhibit CAF-cancer cell interactions for inhibition of metastasis.

## Notch signaling in the TME

### Endothelial cells

Tumor initiation and progression highly depends on the formation of new blood vessels, a process termed as “angiogenesis” to provide new supply of nutrients and oxygen to the rapidly proliferating tumor cells in an increasingly hypoxic TME (Folkman, 2002; Nishida et al., 2006; Lugano et al., 2020). The dynamicity of angiogenesis is largely determined by the interplay of two different kinds of endothelial cells, termed as tip cells and stalk cells (Blanco and Gerhardt, 2013; Lugano et al., 2020). The role of Notch signaling in the regulation of angiogenesis has highly been implicated in several studies (Benedito et al., 2009; Blanco and Gerhardt, 2013; Liu et al., 2014; Banerjee et al., 2015; Kuhnert et al., 2015; Xu Z. et al., 2016; Pitulescu et al., 2017; Akil et al., 2021). An interesting interplay between the Notch ligands, Dll4 and Jag1 has been shown to regulate angiogenesis by regulating the balance between the formation of tip cells and stalk cells (Benedito et al., 2009; Liu et al., 2014; Akil et al., 2021). By using endothelial cell (EC)-specific genetically inducible mouse models, Benedito et al. (2009) demonstrated that the Notch ligand Dll4 inhibits angiogenesis by activating Notch signaling in tip cells, leading to decreased vessel sprouting. Interestingly, another Notch ligand Jag1 inhibits the Dll4-mediated signaling, induces vessel sprouting and further leads to angiogenesis (Benedito et al., 2009). Not surprisingly, the role of this angiogenic crosstalk has been implicated in various human cancers as well (Noguera-Troise et al., 2006; Liu et al., 2014; Banerjee et al., 2015; Kuhnert et al., 2015; Xu Z. et al., 2016; Lugano et al., 2020; Akil et al., 2021). Accordingly, Kuhnert et al. (2015) demonstrated that antibody-mediated blocking of Dll4-Notch signaling in stromal cells of ovarian cancer humanized mouse models. The authors further identified a paracrine signaling between EC-expressing Dll4 and adjacent tumor cell-expressing Notch1, whose inhibition led to increased angiogenesis, with reduced vascular perfusions and demonstrated a potent anti-tumor effect (Kuhnert et al., 2015). Interestingly, the anti-tumor effect of Dll4-blockade was potentiated by combinatorial inhibition of VEGF signaling further providing potential anti-cancer therapeutic opportunities (Kuhnert et al., 2015).

A similar work demonstrated the anti-tumor role of Dll4 blockade in breast cancer mouse xenograft models, where combined treatment of anti-Dll4 antibody in combination with docetaxel led to tumor cell apoptosis, CSC phenotype and reversal of EMT (Xu Z. et al., 2016). In an intriguing study, inhibition of Notch signaling in liver stromal cells of the TME was shown to promote highly vascularized liver metastases of neuroblastoma and breast cancer cells (Banerjee et al., 2015). Mechanistically, this phenomenon was shown to be mediated specifically by Dll-Notch1 signaling, since blockade of the same using N1-decoy constructs led to increased proliferation and sprouting of sinusoidal endothelial cells further causing the development of liver micro metastases (Banerjee et al., 2015). This work aligns with the known pleiotropic nature of Notch signaling, this time demonstrating both a tumor suppressive and oncogenic role with respect to angiogenesis and metastasis. Besides acting alone, Notch has also been reported to collaborate with VEGF for the regulation of angiogenesis, particularly in the specification of tip/stalk cells (Blanco and Gerhardt, 2013; Lugano et al., 2020; Akil et al., 2021). VEGF induces production of Dll4 from tip cells causing activation of Notch in neighboring endothelial cells, ultimately leading to vessel sprouting (Blanco and Gerhardt, 2013; Lugano et al., 2020; Akil et al., 2021). Unsurprisingly, this crosstalk has been implicated in cancer. Cancer cells have been demonstrated to secrete VEGF capable of inducing Dll4 in endothelial cells of the TME, which negatively regulates excessive unproductive sprouting and helps to maintain tumor angiogenesis at a steady rate (Noguera-Troise et al., 2006). This indicates an interesting aspect of the tumor cells trying to maintain a balance between densely sprouted non-functional vasculature and less dense, but well-structured angiogenic vessel, that can be manipulated for anti-angiogenic therapy. Correspondingly, blockade of Dll4 binding to Notch1 significantly decreased tumor growth and vasculature of mouse mammary tumors already resistant to VEGF blockade (Noguera-Troise et al., 2006). Thus, this suggests potential therapeutic benefits of blocking Dll4 in patients resistant to VEGF targeted therapies. The role of Notch signaling has also recently been implicated in the process of endothelial-to-mesenchymal transition (EndoMT) which depicts the transition of endothelial cells into a mesenchymal phenotype and is well articulated in a recent review by Akil et al. (2021).

### Immune cells

The role of Notch signaling pathway in the development of the hematopoietic system and its associated plethora of immune cells including B-cells, T-cells, cells of the myeloid lineage, etc. is beyond the scope of this paper and has extensively been highlighted in a detailed review by Radtke et al. (2010). However, we aim to cover the various aspects of the Notch signaling in various immune cells of the TME and how that corresponds to various cancers. Immune cells of the TME can largely be either tumor-promoting/immune-suppressive, including tumor associated macrophages (TAMs), myeloid-derived suppressor cells (MDSCs), regulatory T-cells (T_regs_) (Lu et al., 2019) or tumor-suppressive/immune-promoting that includes dendritic cells (DCs), cytotoxic T-cells (CD8^+^), natural killer (NK) cells, proinflammatory macrophages (Labani-Motlagh et al., 2020). Accordingly, Notch signaling has been shown to play an important role in regulating the balance between immune-promoting and immune-suppressive TME.

TAMs are known to switch between two polarizing states called M1 (anti-tumorigenic/inflammatory) and M2 (pro-tumorigenic/immune-suppressive) (Caux et al., 2016) and Notch signaling has been shown to tip the balance in favor of an M1 state (Wang et al., 2010; Zhao et al., 2016; Lin et al., 2018). M1 polarized macrophages isolated from mice with melanoma tumors were seen to express increased Notch ligands, receptors and Hes1 (Wang et al., 2010). Intriguingly, Dll4-induced activation of Notch signaling in monocytes polarized them to an M1 state, which could be reversed by using a gamma secretase inhibitor to block Notch signaling. The Notch activated macrophages were also more anti-tumorigenic in nature, with macrophages with genetic deletion of RBPj lacking capacity to activate T-cells (Wang et al., 2010). Constitutive activation of the NICD1 in a transgenic mouse model was shown to inhibit tumor growth, increase M1 macrophage markers and CD8^+^T-cells while decreasing MDSCs (Zhao et al., 2016). This NICD1-mediated effect of M1 polarization was shown to occur via the binding of miR-125a to FIH1 and IRF4, both of which suppressed M2 state and induced an anti-tumorigenic M1 state (Zhao et al., 2016). SIRPα, an Ig family protein involved in suppression of phagocytosis was identified to be repressed by activated Notch signaling via Hes1 (Lin et al., 2018). Interestingly, this downregulation of SIRPα was important for an M1 polarization, and phagocytosis of tumor cells. These results identify Notch signaling activation as a key switch driving an M1 like state in TAMs and provides an avenue for reprogramming M2 TAMs to an M1 state for anti-tumor therapies. The cell- and context-dependent diversity in Notch signal output is also seemingly apparent in this scenario since activated Notch signaling has been shown to recruit pro-tumorigenic TAMs (Shen et al., 2017; Huang et al., 2021). Shen et al. (2017) reported a paracrine crosstalk between TAMs and cancer cells in basal-like breast cancer leading to Notch activation within these cancer cells. Interestingly, the Notch activated cancer cells would be key in the recruitment of pro-tumorigenic TAMs by secretion of IL-1b and CCL2. Clinically, in human basal-like breast cancer tumors, a correlation between Notch activation, TAM infiltration and poor prognosis was observed, evidencing anti-Notch mediated pro-tumorigenic TAM targeting for therapy (Shen et al., 2017). In diffuse large B-cell lymphoma, mutations in CREBBP/EP300 were shown to downregulate negative regulator of Notch signaling, FBXW7 (Huang et al., 2021). The downregulation of FBXW7 concomitantly increased Notch-mediated secretion of CCL2/CSF1 which in turn polarized TAMs to a pro-tumorigenic M2 state. CREBBP/EP300 mutation carrying xenografts demonstrated increased Notch targets Hey1 and Hey2, CCL2 secretion compared to CREBBP/EP300 wild type tumor xenografts (Huang et al., 2021).

NK cells can recognize and proficiently kill cancer cells thus providing an anti-tumorigenic role (Shimasaki et al., 2020). Accordingly, it is important to keep NK cells activated to be able to harness this benefit and Notch has been identified as an important activator of NK cells (DeHart et al., 2005; Kijima et al., 2008). Coculturing of jag2 expressing cells with hematopoietic stem cells has been shown to induce Notch-mediated differentiation into NK cells (DeHart et al., 2005). Jag2 expression on DCs was shown to activate Notch2 in NK cells important for their proliferation and cytolytic activity (Kijima et al., 2008). This jag2-mediated NK cell activation was important for reducing tumor size *in vivo* and this activation could be inhibited by using a GSI against Notch (Kijima et al., 2008). An important component of the tumor immune microenvironment involves the MDSCs which play suppressive roles toward NK-cell and T-cell activity (Law et al., 2020). Tumor cells harness the immune suppressive effects of MDSCs to create an environment suitable for their growth and metastasis (Law et al., 2020). Cancer cells have been observed to induce expression of Jag1/2 in MDSCs via the NF-Kβ pathway and targeting Jag1/2 in MDSCs by use of a neutralizing antibody was shown to inhibit arginase I, iNOS and decreased tumor growth (Sierra et al., 2017). The anti-jag1/2 targeted antibody also led to increased cytotoxic T cell infiltration into tumors which are normally suppressed by MDSCs. Notch signaling has been identified as an important regulator of lactate metabolism to drive the differentiation of M-MDSCs to M1-like TAMs to mediate an anti-tumorigenic response (Zhao et al., 2022). The presence of lactate is an important driver towards a M2-like state which is crucial for tumor cell survival. The authors have demonstrated that activated Notch signaling can induce its target Hes1, which in turn can repress the lactate importer MCT2 thus, reducing lactate accumulation. Lack of lactate was shown to lead to FBXW7-mediated degradation of c-jun and inhibition of COX2 which was important for the differentiation of M-MDSCs to an anti-tumorigenic M1-TAM state (Zhao et al., 2022). Accordingly, activation of NICD in mice led to repressed tumor growth and in clinical settings, higher grade lung biopsies were shown to have less of Hes1^+^CD^68+^ cells indicative of an immunosuppressive TME (Zhao et al., 2022).

## Notch signaling in CAFs

**FIGURE 2:**
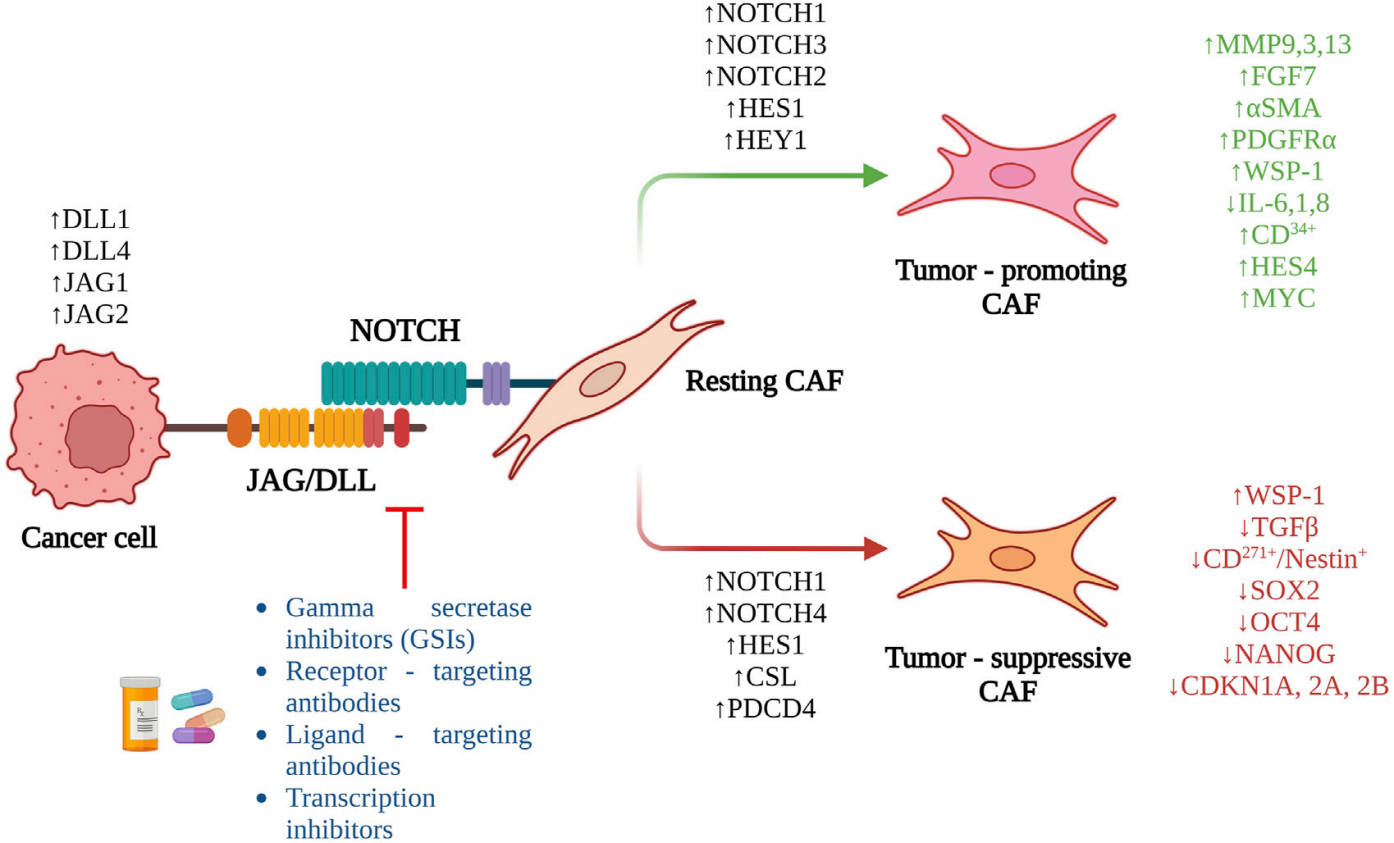
Overview of the role of Notch signaling in CAF activation and the potential therapeutic interventions targeting the pathway.

We have discussed previously the pleotropic roles of Notch signaling activation in metastasis. However, even though CAFs are an important and abundant component of the TME, there has been limited research investigating the role of Notch in CAFs (Figure 2). Hu et al. (2012) were one of the earliest to demonstrate the pro-tumorigenic effect of mesenchymal deletion of Notch pathway component, CSL in mouse skin. Targeted deletion of CSL gene in mesenchymal cells of mice led to development of keratinocyte tumors similar in histology as that of squamous cell carcinoma, within 2–4 months of birth. Mesenchymal deletion of CSL led to increased immune infiltration, recruitment of macrophages, dermal hyperplasia, and keratinocyte proliferation. Interestingly, this tumorigenic effect could be reduced by treating these mice at birth and not after, with an anti-inflammatory agent strongly indicating that the pro-tumorigenic effect of mesenchymal CSL deletion was driven by increased inflammation (Hu et al., 2012). Intriguingly, mixing of CSL^−/−^ fibroblasts with SCC keratinocyte lines and injection into immunocompromised mice led to development of larger, less-differentiated and highly proliferative tumors compared to control fibroblasts (Hu et al., 2012). The CSL^−/−^ fibroblasts demonstrated increase in CAF markers, αSMA, PDGFRα, growth factor genes, FGF7, IGF2, and matrix metalloproteases, MMP3, 9, and 13. By mixing normal keratinocytes with CSL^−/−^ fibroblasts and injecting them in mice, the authors observed signs of cellular atypia, loss of cellular differentiation, and uneven basement membrane (Hu et al., 2012). Mechanistic insights revealed a Notch-mediated negative control of AP1 signaling components, c-jun and c-fos. Concomitantly, additional inhibition of c-jun and fos in CSL^−/−^ fibroblasts led to decrease in tumorigenesis when mixed with SCC cells and injected into mice. Clinically, skin samples from patients with pre-SCC lesions demonstrated a similar inverse relationship between Notch signaling and its negatively controlled effectors such as, AP-1 and CAF markers (Hu et al., 2012).

This group further reported important mechanistic findings pertaining to the role of CSL in the tumor-suppressive effects of dermal CAFs (Procopio et al., 2015; Jo et al., 2016). Procopio et al. (2015) reported that the CSL^−/−^ dermal fibroblasts displayed increased senescence-associated morphology and reduced proliferation. This was in line with silencing effect of CSL in human dermal fibroblasts which demonstrated increased expression of senescence determinants, CDKN2B, 2A and 1A while also increasing expression of CAF marker genes, such as αSMA (Procopio et al., 2015). Binding and protein-protein interaction studies demonstrated interaction between p53 and CSL, which was crucial for p53 activity since CSL silencing increased p53 transcriptional activity and *vice versa* (Procopio et al., 2015). Importantly, analysis of micro dissected fibroblasts from SCC patients’ skin samples showed downmodulation of CSL, p53 and CDKN1A compared to normal skin samples. Further *in vivo* mechanistic experiments implicated a two-way regulation of normal fibroblast to CAF transition, involving downregulation of CSL and p53. Initial downregulation of both CSL and p53 are important in the expression of senescence genes and suppression of CAF genes that prevents acquisition of a CAF state. Importantly, neoplastic transformation of cancer cells can trigger a disruption in this negative CAF regulation via secretion of growth factors or cytokines leading to the activation of CAF genes and loss of senescence effector genes (Procopio et al., 2015). This co-evolutionary crosstalk thus, is important for cancer-stromal expansion and provides a therapeutic avenue targeting CSL and p53 in dermal CAFs.

Jo *et al*, identified a novel association between the tumor suppressor PDCD4 and CSL in human dermal fibroblasts (Jo et al., 2016). Importantly, PDCD4 was shown to bind to promoter regions of CAF genes in a CSL dependent manner and repress activation of CAF genes, senescence effector genes and Notch signaling targets (Jo et al., 2016). Functionally, tumors formed by SCC cells admixed with PDCD4 silenced human dermal fibroblasts led to larger tumors, increased cancer cell proliferation and decreased differentiation compared to control human dermal fibroblasts *in vivo* (Jo et al., 2016). Importantly, the pro- or anti-tumorigenic roles of Notch signaling in CAFs have been shown to vary based on the specific Notch receptors driving the process. Interestingly, the same Notch receptor can also display different roles in different cancer types. Accordingly, NOTCH1 signaling in CAFs has largely been shown to have a tumor suppressive effect (Shao et al., 2011; Shao H. et al., 2015; Shao et al., 2021) with some reports indicating its pro-tumorigenic effect as well (Hoare et al., 2016; Nabet et al., 2017). By combining tissue microarray and IF staining, Shao et al. (2021) demonstrated that melanoma associated fibroblasts (MAFs) at various stages of melanoma displayed very low activity of HES1 when compared to that of adjacent/non-adjacent normal skin fibroblasts. Notch receptors 1 and 4 along with downstream targets were observed to be downregulated in MAFs both at an RNA and protein level compared to normal human skin and dermal fibroblasts. Interestingly, overexpression of activated NOTCH1 in MAFs led to reduced proliferation and increased apoptosis of the MAFs in addition to inhibiting growth of melanoma cells in cocultures (Shao et al., 2021). *In vivo*, tumors comprised of co-grafted active NOTCH1 overexpressing MAFs and melanoma cells demonstrated smaller volumes, weight and intriguingly, reduced blood vessel densities compared to controls (Shao et al., 2021). By creating an FSP^+^ fibroblast specific GOF^Notch1^ mice and inoculating melanoma cells in them, the authors demonstrated reduced melanoma tumor growth in these mice compared to control (Shao H. et al., 2015). Interestingly, melanoma cells adjacent to these CAFs were less proliferative compared to ones at a distance. Tumor tissue sections from GOF^Notch1^ mice displayed less invasion of melanoma cells and less proliferation of CAFs and the reverse was also true. Even though tumors from LOF^Notch1^ mice did not demonstrate significant differences in volume, they were more invasive and contained more proliferative CAFs (Shao H. et al., 2015). Mechanistically, the effect of activated NOTCH1 in fibroblasts to suppress melanoma growth and angiogenesis has been shown to be mediated by the secretion of wnt-induced secreted protein-1 (WSP-1) and identifying the NOTCH1-mediated WISP1 as an important player (Shao et al., 2011).

Cancer cells are known to reprogram CAFs via paracrine secretion of various factors. Accordingly, in an interesting study, exposure of CAFs to apoptotic lung cancer cells were found to inhibit CAF invasion and migration via the NOTCH1-WISP1-TGFβ signaling pathway (Kim et al., 2022). Additionally, these exposed CAFs were shown to display a similar paracrine inhibitory effect on lung cancer cells mediated by secretion of WISP-1. DII1 expressed by apoptotic lung cancer cells triggered the NOTCH1-WISP1 axis in CAFs, inhibited their activation, and enabled them to inhibit tumor-migration and invasion via an efferocytic activity (Kim et al., 2022). Importantly, *in vivo*, injection of apoptotic lung cancer cells or recombinant WISP-1 could induce this signaling axis to inhibit CAF activation and reduction in metastatic tumors (Kim et al., 2022). Other mechanisms employed by CAFs to induced tumorigenicity include the regulation and induction of cancer stem cells (CSCs). In this aspect, loss of active NOTCH1 signaling in mesenchymal stem cell derived fibroblasts (MSC-DFs) have been implicated to induce CD^271+^/Nestin^+^ melanoma initiating cells along with enhanced spheroid formation ability (Du et al., 2019). These Notch1^−/−^CAF-induced CD^271+^ melanoma initiating cells also exhibited increased stem-markers sox2, oct4 and nanog; proliferative capacity, invasiveness, and enhanced lung metastasis *in vivo* (Du et al., 2019). These studies demonstrated the tumor-inhibiting role of activated Notch1 in CAFs in the context of melanoma and lung cancer, thus providing an important therapeutic avenue by utilizing and inducing NOTCH1.

Given the context-dependency and pleotropicity of activated Notch signaling, NOTCH1 in CAFs has also been shown to have important tumor-promoting roles. Nabet et al. (2017) reported the activation of stromal fibroblasts via Notch-MYC signaling induced by the co-culture with interferon stimulated gene-responding (ISG-R) breast cancer cells, not observed in co-cultures with ISG-non-responding (ISG-NR) cancer cells. Upregulation of active NICD1 and its downstream target, MYC was observed in fibroblasts cocultured with ISG-R breast cancer cells. This activation of NOTCH1-MYC signaling produced unshielded exosomal RNA, RN7SL1 which further induced ISGs in an autocrine manner in fibroblasts as well as in a paracrine manner in the breast cancer cells (Nabet et al., 2017). Importantly, the unshielded exosomal RN7SL1 obtained from tumor-stromal cocultures enhanced tumor growth and metastasis in breast cancer xenografts. Clinically, patient isolated cancer associated fibroblasts exhibited similar upregulation of NOTCH1-MYC signaling along with production of unshielded exosomal RN7SL1, thus providing therapeutic relevance (Nabet et al., 2017). In another study, the role of NOTCH1 was investigated and found to be pleotropic for the regulation of senescence in human diploid fibroblasts (HDFs) (Hoare et al., 2016). Interestingly, the NOTCH1 mediated signaling was found to positive regulate TGFβ whereas, negative regulate pro-inflammatory cytokines IL-6, 1 and 8, despite all of them being components of a senescence associated secretory phenotype (SASP) (Hoare et al., 2016). Intriguingly, non-senescent fibroblasts cocultured with NOTCH1-induced senescent fibroblasts also gained senescent phenotype and upregulated TGFβ pathway components that could be maintained even after the inducing cells were removed. This NICD1-mediated induction of senescence was shown to be mediated by repression of the transcription factor C/EBPB and functionally inhibited immune infiltration and led to accelerated tumor formation *in vivo* (Hoare et al., 2016). This study provides a NOTCH1-targeted therapeutic aspect for cancer since senescent cells have been reported to be important for tumor progression.

The role of NOTCH2 pathway in fibroblasts has been reported in ductal carcinoma *in situ* (DCIS). Strell et al. (2019) reported the induction of NOTCH2 and its downstream targets HES1 and HEY1 in a specific fibroblast subset defined as PDGFRα ^(low)^/PDGFRβ ^(high)^ upon coculturing with DCIS cells expressing JAG1. Loss of JAG1 from the DCIS cells or loss of NOTCH2 from the PDGFRα ^(low)^/PDGFRβ ^(high)^ fibroblasts abrogated the induction of downstream targets along with downregulation of PDGFRβ expression. In human DCIS clinical samples, an inverse correlation was observed between HES1 and PDGFRα expression, and in patient cohorts, the PDGFRα ^(low)^/PDGFRβ ^(high)^ fibroblast subset exhibited poor prognosis, further providing evidence for the importance of NOTCH2 in this specific fibroblast subset for DCIS progression (Strell et al., 2019). Xue et al. (2021) reported the upregulation of Notch ligands Jag1 and Jag2 in metastatic lung cancer cells lacking miR-200. The ligand expressing metastatic lung cancer cells were shown to induce Notch signaling in CAFs via NOTCH1 and NOTCH3 in organoid cocultures enabling invasive features that could be ablated by knockdown of the ligands or overexpression of miR-200 (Xue et al., 2021). Human lung patient metastatic tumors stained for active NICD1 and NICD3 showed strong enrichment in CAFs compared to primary tumors (Xue et al., 2021), further reiterating the role of active Notch signaling in CAFs for tumor progression and metastasis. This makes it more interesting since either of the two ligands Jag1 or Jag2 could be involved in crosstalks with NOTCH1/NOTCH2 rendering different possibilities.

Apart from NOTCH1 and NOTCH2, NOTCH3 has also been reported to be important for CAF activation in pancreatic and oral squamous cell carcinomas (Kayamori et al., 2016; Song and Zhang, 2018). PDAC tumors are characterized by the presence of a desmoplastic stroma, meaning enrichment of CAFs and secreted ECM proteins. Accordingly, activated pancreatic stellate cells (PaSCs) which are myofibroblastic in nature have been shown to have upregulated NOTCH1,2 and 3 with activated NOTCH3 being exclusively expressed in αSMA^+^ PaSCs (Song and Zhang, 2018). Interestingly, the expression of NOTCH4 was either absent or downregulated. Knockdown of NOTCH3 was effective in reversing activated PaSCs into quiescent state indicated by the accumulation of lipid droplets, in addition to downregulating activated PaSC markers such as αSMA, collagen I and fibronectin (Song and Zhang, 2018). PaSCs with inhibited NOTCH3 were less proliferative and migratory, and also were less effective in inducing migration of pancreatic cancer cells (Song and Zhang, 2018). Analysis of human oral squamous cell carcinoma patient samples rendered presence of NOTCH3^+^αSMA^+^CAFs in the vicinity of cancer nests leading to poor overall survival rate (Kayamori et al., 2016). Coculturing of oral squamous carcinoma cells with fibroblasts led to induction of both NOTCH3 and αSMA, which interestingly localized in close vicinity of the tumor nests further indicating contact-mediated induction of NOTCH3. Additionally, only contact mediated coculturing of cancer cells could specifically induce NOTCH3, not observed when the cells were separated by a transwell, removing the probabilities of paracrine mechanisms (Kayamori et al., 2016). Immunofluorescence staining of tumor samples demonstrated increased CD^34+^ micro vessels in NOTCH3^+^CAF containing samples indicative of angiogenesis. *In vitro*, cocultures using NOTCH3^+^CAFs increased CD^31+^endothelial cell containing vessels with enhanced tube formation, ablated upon the knockdown of NOTCH3 (Kayamori et al., 2016). These results demonstrate the pro-angiogenic functionality of NOTCH3^+^CAFs in oral squamous cell carcinoma providing therapeutic opportunities either by the targeting of these CAFs alone or in combination with existing angiogenesis inhibitors.

Cancer cell-CAF crosstalk governed by cellular signaling pathways are known to reprogram and drive functional heterogeneity of CAFs. Accordingly, single cell RNA sequencing analysis of Thy^+^CAFs isolated from lung adenocarcinoma mice model demonstrated existence of two distinct clusters, one of which showed enrichment in Notch signaling pathway (Bota-Rabassedas et al., 2021). Interestingly, this characteristic cluster signature could be recapitulated when the Thy^+^CAFs were cocultured will highly epithelial but poorly metastatic lung adenocarcinoma cells indicating that the these highly epithelial cells could be driving ligand induced Notch signaling which might be lost when they became more mesenchymal in nature (Bota-Rabassedas et al., 2021). In a study performed to understand CAF heterogeneity in breast cancer metastasis, Pelon and colleagues identified enrichment of two CAF subsets (S1 and S4) in metastatic lymph nodes as we have described before (Pelon et al., 2020). However, of interest is the fact that CAF-S4 had significant upregulation of NOTCH receptors 1-3 and demonstrated matrix remodeling functionality. Interestingly, use of a gamma-secretase inhibitor did not change the viability of these CAFs but significantly hampered their collagen contracting function, further indicating requirement of NOTCH for CAF functionality rather than survival (Pelon et al., 2020). The CAF-S4 was key for inducing cancer cell invasion and motility in 3D, which again was reduced upon treatment with the inhibitor. Prognostically, only patients with enriched stromal and CAF-S4 content in the lymph nodes displayed poorest OS and increased liver metastases, further providing clinical relevance for NOTCH-targeting in CAFs (Pelon et al., 2020). An important pan-cancer single cell RNA sequencing analysis comprising of the stromal cells from 10 different solid tumors identified significant enrichment of NOTCH3 and HES4 in CAFs compared to normal fibroblasts (Luo et al., 2022). Using trajectory analyses, the authors defined three major CAF states, and gene fluctuation analyses revealed that CAF state 2, defined to be similar to myofibroblastic CAFs, had enriched NOTCH3 expression compared to the other two states (Luo et al., 2022). Interestingly, CAF states 2 and 3 (inflammatory/adipogenic CAFs) were shown to mediate crosstalks with tumor endothelial cells notably via NOTCH1/2/3_JAG1/2/DLL4 pathways reiterating the role of NOTCH signaling in CAF-mediated angiogenesis (Luo et al., 2022). Unsurprisingly, this NOTCH-mediated crosstalk was also observed between CAF states 2 and 3 and tumor epithelial cell. All these results that we have summed up critically identify the role of NOTCH signaling pathway in CAFs, in various solid tumors and metastases. Of note is the fact that the role of NOTCH in CAFs can either be that of tumor-suppressive or tumor-promoting based on the type of cancer, or the particular NOTCH receptor involved.

## Targeting Notch signaling in cancer

As we have described above, the Notch signaling pathway plays critical roles in development and oncogenesis. Importantly, it has roles in the pro-tumorigenic ability of cancer cells along with stromal cells. This makes the Notch signaling pathway a hot target for the development of potential anti-cancer therapeutics. Accordingly, several small molecule inhibitors, neutralizing antibodies, signal agonists and transcriptional inhibitors have been developed to target various steps to either block or activate this pathway. We have summarized some of these molecules, their mechanism of action and their performance in clinical trials:

### Gamma-secretase inhibitors (GSIs)

The gamma secretase complex, as described above, plays crucial role in the activation of the notch signaling pathway by mediating the S3 cleavage process of the notch receptor to produce the intracellular domain. Accordingly, this complex has been a key target for the development of small molecules named broadly as GSIs. GSIs were initially used as treatments for Alzheimer’s disease since they prevent the production of beta amyloids. An oral GSI named MK-0752, developed by Merck was one of the earliest ones to be used in a phase I clinical study for patients with relapsed T-ALL and other leukemias (NCT00100152) but this study was terminated due to severe diarrhea as dose-limiting toxicity (DLT) in participants. The maximally tolerated dose (MTD) and DLT of MK-0752 was assessed in a phase I clinical trial involving children with refractory CNS tumors showing lack of GI toxicity as observed in adults (NCT00572182) (Fouladi et al., 2011). However, tumor progression was not halted in most patients after receiving one or two courses and was later discontinued. In a large phase I study involving 103 patients with various solid tumors, the DLT and MTD of MK-0752 were assessed (NCT00106145) (Krop et al., 2012). These published results reported that one patient with anaplastic astrocytoma exhibiting complete response of more than a year and 10 others with various gliomas exhibiting stable disease for more than a year (Krop et al., 2012). Even though the anti-tumor activity of MK-0752 was modest in patients with other cancers, it was notable in patients with brain tumors involved in this study thus setting up a platform for further research and trials (Krop et al., 2012). A phase I clinical trial to assess the effectivity of MK-0752 in combination with gemcitabine hydrochloride for the treatment of patients with stage III and IV pancreatic cancer where surgical intervention is not possible is also currently underway (NCT01098344).

Another potent and prominent GSI called nirogacestat or PF-03084014 has been used in multiple phase I and II clinical trials for various tumor types. A phase II clinical trial involving PF-03084014 in patients with aggressive fibromatosis/desmoid tumors (NCT01981551) rendered 29% of patients exhibiting partial responses of more than 2 years and another 29% with prolonged stable disease, and tolerable toxicities rendering the drug a promising target for desmoid tumors (Kummar et al., 2017). Concomitantly, there is an actively recruiting phase III study for the evaluation of nirogacestat in patients with desmoid tumors (NCT03785964). There are multiple ongoing phase I and II clinical studies pertaining to nirogacestat including, a phase II study in ovarian granulosa tumors (NCT05348356), a phase II umbrella study combining elranatamab and nirogacestat in multiple myeloma (NCT05090566), a phase I combination study of belantamab mafodotin and nirogacestat in people with relapsed/refractory multiple myeloma (NCT05556798) and a phase I study combining allogenic CAR T-cells with or without nirogacestat in refractory multiple myeloma patients (NCT04093596). A phase I clinical trial using the GSI, RO4929097 was carried out in patients with locally advanced or refractory metastatic solid tumors (NCT00532090) which reported common toxicities such as fatigue, thrombocytopenia, fever, in addition to one patient with DLT (grade 3 hypophosphatemia) and two patients with grade 3 pruritus (Tolcher et al., 2012). Evaluation of anti-tumor activity demonstrated one patient with colorectal adenocarcinoma displaying partial response, mixed response in a patient with sarcoma and one nearly complete FDG-PET response in a melanoma patient (Tolcher et al., 2012). A phase II study in stage IV metastatic melanoma patients was published with RO4929097, which was later terminated (NCT01120275) due to insufficient response. Only one out of 32 patients had a partial response and 8 patients with stable disease (Lee et al., 2015). In a phase 0/I trial involving combination treatments of RO492909, temozolomide and radiation therapy (NCT01119599) in patients with newly diagnosed malignant gliomas rendered promising results such as well tolerability and not DLT, along with reduced Notch activity and CD^133+^ cancer initiating cells (Xu R. et al., 2016).

The first human study involving the oral GSI, LY3039478 also known as crenigacestat showed promising results in patients with advanced or metastatic cancer (NCT01695005). Patients with breast carcinoma, adenoid cystic carcinoma and leiomyosarcoma exhibited tumor necrosis or tumor shrinkage with tolerable toxicities (Massard et al., 2018). Crenigacestat was also combined with dexamethasone in a phase I clinical trial in patients with relapsed/refractory T-ALL/T-LBL (NCT02518113) resulting in one patient with T-ALL having a complete response, six patients achieving stable diseases. Despite the efficacy, overall GI toxicities were high in the patients, requiring further dose evaluation (Borthakur et al., 2021). These trial results suggest that only nirogacestat or PF-03084014 stands out to be the most promising GSIs, with others being less efficacious. It is not surprising that GSIs have still not made it to FDA approvals given their toxicities in patients. However, the current ongoing trials with nirogacestat shows a promising hope for the future.

### Notch receptor-targeting antibodies

Antagonistic antibodies targeted against specific NOTCH receptors have been developed and used in clinical trials to prevent binding of ligands and inhibiting downstream signal activation. A monoclonal antibody specifically designed to target NOTCH1, called brontictuzumab has been evaluated in a phase I dose-escalation and dose-expansion clinical trial in patients with selected refractory solid tumors (NCT01778439) (Ferrarotto et al., 2018). Results reported DLTs in three patients with the most common adverse effects to be diarrhea, nausea, and fatigue. Six patients experienced benefits with two patients with adenoid cystic carcinoma having unconfirmed partial response and four having stabilized disease for more than 6 months out of which three had adenoid cystic carcinoma (Ferrarotto et al., 2018). Other clinical trials involving Brontictuzumab includes a phase I dose-escalation study in patients with lymphoid malignancies (NCT01703572), and a phase 1b dose-escalation study in a combination with chemotherapy for subjects with previously treated metastatic colorectal cancer (NCT03031691). A novel antibody targeting both Notch2 and Notch3 called tarextumab or OMP-59R5, developed by Yen et al. (2015), was evaluated in a phase II clinical trial as a combination therapy with nab-paclitaxel and gemtacibine (NCT01647828) in patients with metastatic PDAC (Hu Z. I. et al., 2019). However, strikingly, tarextumab treated patients exhibited worse PFS, no improvements in OS and diarrhea as side effects. In a phase I dose-escalation trial involving patients with solid tumors (NCT01778439), tarextumab was shown to be tolerable with 9 subjects exhibiting stable disease (Smith et al., 2019). A phase I dose-escalation trial using a specific Notch3 targeted antibody-drug conjugate (ADC) called PF-06650808 was performed in patients with breast and other advanced solid tumors (NCT02129205). This study reported well tolerability of PF-06650808 among patients with the most common toxicities being fatigue and loss of appetite (Rosen et al., 2020). 5 subjects showed partial responses and 16 patients exhibiting stable disease (Rosen et al., 2020).

### Ligand-targeting antibodies

Similar to the use of antibodies or ADCs to target notch receptors, notch ligands have also been targeted using similar strategies, the most prominent targets being DLL3 and DLL4. An ADC called rovalpituzumab tesirine (Rova-T) developed to target DLL3 has been evaluated in a phase I study as a frontline mono therapy or in combinations with cisplatin and etoposide for patients with extensive-stage SCLC (NCT02819999). This study reported no added benefit from Rova-T (Hann et al., 2021). Other Rova-T clinical trials include a phase III evaluation of Rova-T as maintenance therapy for SCLC patients after treatment with platinum therapy (NCT03033511) terminated early due to lack of survival benefit (Johnson et al., 2021) and a phase III study comparing efficacy of Rova-T vs. topotecan as a second line therapy for subjects with DLL3-SCLC (NCT03061812), which again was prematurely terminated due to worse OS in the subjects receiving Rova-T (Blackhall et al., 2021). An actively recruiting phase 1/2 trial assessing HPN328, a trispecific T-cell engager targeting DLL3 for patients with DLL3-SCLC has shown promising interim results (NCT04471727) with tumor shrinkage and one confirmed partial response. Efficacy of a monoclonal antibody targeting DLL4 called Enoticumab (REGN421) was assessed in a phase I trial for patients with advanced solid tumors (NCT00871559), where patients exhibited drug tolerability, 2 partial responses and 16 with stable diseases (Chiorean et al., 2015). A bispecific antibody against DLL4 and VEGF called navicixizumab showed promising results in a phase 1a clinical study (NCT02298387) with 4 patients exhibiting partial responses, 17 with stable disease and 19 patients with reduction in size of lesions (Jimeno et al., 2019). A phase 1b study combining navicixizumab with paclitaxel in patients with platinum resistant ovarian cancer (NCT03030287) demonstrated tolerability and durable responses in patients heavily treated priorly, suggesting potential clinical benefit (Fu et al., 2022).

### Transcription inhibitors

These are designed specifically to inhibit the formation of the Notch ternary complex (NTC) comprising of CSL/RBPj, NICD and MAML1. Accordingly, the earliest report utilizing this targeting aspect was submitted by Astudillo et al. (2016) identifying a novel compound IMR-1 (inhibitor of mastermind 1 recruitment) which prevents the binding of MAML1 to the NTC. IMR-1 inhibited colony formation of cancer cells *in vitro*, in addition to showing promising inhibitory effect of mice tumors *in vivo*. A second inhibitor called RIN-1 (RBPj inhibitor-1) with a mechanism of action directed towards the inhibition of RBPj and SHARP interaction was reported by Hurtado et al. (2019). RIN-1 demonstrated similar effects as that observed with RBPJ silencing and could inhibit the proliferation of cancer cells *in vitro* (Hurtado et al., 2019). A more recent orally active small molecule named CB-103 was identified as a pan-Notch inhibitor with a mechanistic prevention of the NTC formation (Lehal et al., 2020). The most intriguing aspect of CB-103 apart from its potent anti-tumorigenic effect was the fact that CB-103 treatment in mice did not produce the GI toxicities observed with GSIs, making it a more promising therapeutic agent for Notch targeting (Lehal et al., 2020). Accordingly, an actively recruiting phase I/II study of CB-103 alone or in combination with venetoclax in patients with NOTCH adenoid cystic carcinoma is in process (NCT05774899).

## Targeting Notch signaling in the TME

Most of the approaches described above have been used specifically to eradicate cancer cells by targeting various notch components. However, recently there has been a paradigm shift in the development of cancer therapeutics by targeting different stromal cells of the TME, given that they play a major role in tumor progression as well. A major part of our paper has focused on describing the various aspects of notch in different stromal cells of the TME and accordingly, we have also aimed at summarizing evidence targeting notch in the TME. The tumor and its microenvironment mediated induction of immune checkpoint inhibitors such as PD-L1 have been well documented to play a crucial role towards immune suppression and resistance to immunotherapy. Bottcher et al. (2021) demonstrated that in CLL, cross-talk between bone marrow derived stromal cells and CLL cells upregulates PD-L1 and mechanistically this is mediated via the Notch signaling. The authors reported that bone marrow derived stromal cells express increased notch ligands Jag1, Jag2, Dll1 and Dll3 critical for the induction of Notch-Myc-EZH2 axis mediated PD-L1 expression in CLL cells (Bottcher et al., 2021). This proves to be an important mechanism of T-cell suppression. Interestingly, they also demonstrated that application of immunomodulatory drugs (IMiDs) could downregulate these Notch ligands in the stromal cells further reducing their capability of inducing Notch in CLL cells. This study shows different avenues of exploring this axis for therapeutic targeting of CLL. Combined inhibition of notch ligands in stromal cells and notch pathway components in the cancer cells can prevent cross-talk mediated induction of PD-L1 and potentially resensitize tumors to checkpoint inhibitors. This can also alternatively be approached by using IMiDs to downregulate the ligands in the stromal cells as shown by the authors (Bottcher et al., 2021). Finally, a combination of Notch inhibitors such as GSIs with immune checkpoint inhibitors can also be utilized as a mechanism of overcoming immune-checkpoint inhibitor resistance in CLL.

We have previously described how Notch signaling can suppress anti-tumor immunity via regulation of MDSCs, and accordingly, targeting this aspect has been shown to be important therapeutically. In a study by Sierra et al. (2017) the therapeutic activity of a humanized anti-jagged1/2 blocking antibody CTX014 was assessed in tumor mice models. CTX014 was shown to have an anti-tumor effect via accumulation of antitumor MDSC-LC and cytotoxic CD8^+^ T cells (Sierra et al., 2017). Interestingly, CTX014 was shown to induce Notch1 and Hes1 in these CD8^+^T cells which is unsurprising given the pleotropicity of Notch. Upregulation of Jagged1 and 2 in MDSCs was largely mediated by cross-talk with cancer cells inducing NFkB-p65-mediated direct binding of Jag1 and 2 promoters and their increased expression (Sierra et al., 2017). This study demonstrates how tumor cells can induce notch ligands in MDSCs, critical for the suppression of T cell mediated anti-tumor immunity. Important therapeutic implications from this report includes potential combinatorial treatments of CTX014 and NFkB-p65 inhibitors for anti-tumor immunity. Other reports have also bolstered the potential use of Notch inhibition for the generation of anti-tumor immunity. By using a GSI, DAPT, Mao et al. (2018) demonstrated that Notch1 inhibition led to reduced M-MDSCs and PMN-MDSCs in draining lymph nodes, spleen, and tumors of HNSCC bearing mice. Intriguingly, treatment of mice with DAPT *in vivo* significantly decreased expression of major checkpoint molecules, namely, PD1, TIM3, CTLA4 and LAG3 in circulating T cells, while at the same time increasing CD8^+^IFN-γ^+^ T cells in the tumors (Mao et al., 2018). This has important clinical relevance since the authors also demonstrated a strong correlation between Notch1 and immunosuppressive cells such as CD^68+^CD^163+^TAMs and immune checkpoint molecules in patient HNSCC tumor samples. Notch target Hes1, in addition demonstrated positive correlation with Foxp3^+^T_regs_, not seen with Notch1, further indicating potential role of other notch receptors for Hes1 induction (Mao et al., 2018).

The role of endothelial cells expressing Dll4-mediated Notch signaling in ovarian cancer has been discussed in detail, and this has also been utilized as a potentially therapeutic approach by Kuhnert et al. (2015). Zheng et al. (2017) reported the development of an anti-Jagged1 therapeutic antibody 15D11 that they used to target breast cancer bone metastasis in xenograft mice model. Pre-injection of 15D11 before tumor cell injection, followed by twice a week treatment led to significant reduction of bone metastasis compared to IgG treatment. This therapeutic effect of 15D11 was also equally achieved in reducing already established bone metastasis (Zheng et al., 2017). The reduction in metastasis was shown to be mediated not via reduced angiogenesis by via reduction in jagged1-dependent osteoclast differentiation and osteoclastogenesis. Intriguingly, the authors also observed a synergistic effect of 15D11 and paclitaxel, a standard chemotherapeutic agent in reducing bone metastasis (Zheng et al., 2017). Kangsamaksin et al. (2015) took a different and unique approach by designing Notch1 decoys comprising of Notch inhibitors fused to human IgG Fc, capable of selectively inhibiting different classes of notch ligands. It is known that Notch signaling has pleotropic and context-dependent signaling outcomes and in agreement to that, the authors demonstrated that inhibition of Jag1/Jag2-Notch1 interaction had a different mechanistic anti-tumor output compared to Dll1/Dll4-Notch1 inhibition (Kangsamaksin et al., 2015). These Notch1 decoys affected tumor angiogenesis via distinct mechanisms where Dll1/Dll4-Notch1 inhibition led to hyper sprouting with reduced tumor vessel perfusions and Jag1/Jag2-Notch1 inhibition reduced angiogenesis and vessel perfusion via increased sVEGFR-1 and disruption of pericyte-endothelium association (Kangsamaksin et al., 2015). This approach has demonstrated well tolerance with minor hepatic toxicities in mice, suggesting their potential use for overcoming the gastrointestinal toxicities observed with the use of GSIs, although clinical trials await. This also sets the stage for developing other notch receptor specific decoys directed towards inhibiting unique ligand-receptor combinations in specific tumor types.

There are other reports demonstrating the potential of targeting components of notch signaling in cells of the TME, notably endothelial cells and immune cells. Given the pertinent functionality of Notch signaling in other stromal cells of the TME such as CAFs, it will be important to understand the specificities and relevance of unique ligand-receptor combination for the creation of a tumor-promoting or a hostile TME. This will enable us to design TME-targeted therapeutics to either induce or inhibit Notch signaling, in addition to strategizing a ligand/receptor specific inhibitory approach in hopes of overcoming GSI-mediated toxicities and pushing for Notch-therapeutics for the clinical use of cancer treatment.

## Conclusion and future perspectives

In conclusion, Notch signaling has been widely studied in the context of cancer cells with a focus on its role in cancer initiation, progression, and spread. However, our knowledge of the role of notch signaling in the TME, especially in CAFs, remains limited. While recent reports have implicated the role of Notch activation in CAFs, and how that can influence tumor progression further studies are needed to decipher the specific Notch pathways involved, their downstream effects, the interplay between the different pathways, and the overall effects on the CAF phenotype. Moreover, it is now well recognized that CAFs, like cancer cells, are a heterogenous population. Therefore, it will be desirable to study the potential role of specific Notch pathways in specific subpopulations of CAFs. Finally, the potential cellular partners involved in the crosstalk, whether it constitutes homotypic or heterotypic signaling, need to be studied using appropriate 3D culture models or patient specimen. It would be desirable to understand the reciprocity of such signaling and their consequences. With the emergence of single cell multiomics and spatial transcriptomics, such questions can be effectively answered using appropriate models. This can help in identifying therapies that can target Notch pathways in the TME and potentially normalize it (Figure 2). Finally, metastasis is responsible for the vast majority of cancer related deaths. Therefore, targeting Notch may emerge as an effective therapy for metastasis as it can simultaneously target the cancer cells and potentially normalize the TME.
